# Intensive spa and exercise therapy program for returning to work for low back pain patients: a randomized controlled trial

**DOI:** 10.1038/s41598-017-18311-z

**Published:** 2017-12-20

**Authors:** Christelle Nguyen, Isabelle Boutron, Christopher Rein, Gabriel Baron, Katherine Sanchez, Clémence Palazzo, Arnaud Dupeyron, Jean-Max Tessier, Emmanuel Coudeyre, Bénédicte Eschalier, Romain Forestier, Christian-François Roques-Latrille, Ygal Attal, Marie-Martine Lefèvre-Colau, François Rannou, Serge Poiraudeau

**Affiliations:** 1 Sorbonne Paris Cité, Université Paris Descartes, Faculté de Médecine, 75006 Paris, France; 2 AP-HP, Hôpitaux Universitaires Paris Centre, Groupe Hospitalier Cochin, Service de Rééducation et de Réadaptation de l’Appareil Locomoteur et des Pathologies du Rachis, 75014 Paris, France; 3 INSERM UMR 1124, Laboratoire de Pharmacologie, Toxicologie et Signalisation Cellulaire, Faculté des Sciences Fondamentales et Biomédicales, UFR Biomédicale des Saints-Pères, 75006 Paris, France; 4 AP-HP, Hôpital Hôtel-Dieu, Centre d’Épidémiologie Clinique, 75004 Paris, France; 5 INSERM UMR 1153, Centre de Recherche Épidémiologie et Statistique Paris Sorbonne Cité, METHODS Team, 75004 Paris, France; 6 INSERM UMR 1153, Centre de Recherche Épidémiologie et Statistique Paris Sorbonne Cité, ECaMO Team, 75004 Paris, France; 7 Institut Fédératif de Recherche sur le Handicap, 75013 Paris, France; 8 Université de Montpellier 1, Groupe Hospitalier et Universitaire Carémeau, Fédération de Médecine Physique et de Réadaptation, 30000 Nîmes, France; 9 Centre Thermal, 40100 Dax, France; 10 Centre Hospitalo-Universitaire de Clermont-Ferrand, Service de Médecine Physique et de Réadaptation, INRA, Université Clermont-Auvergne, 63000 Clermont, Ferrand France; 11 Centre de recherche rhumatologique et thermale, 15, avenue Charles-de-Gaulle, 73100 Aix-Les-Bains, France; 12 Université Toulouse-Sabatier, 31000 Toulouse, France; 13 Rue Victor Hugo, 73000 Chambéry, France

## Abstract

We aimed to determine whether a 5-day intensive inpatient spa and exercise therapy and educational program is more effective than usual care in improving the rate of returning to work at 1 year for patients with subacute and chronic low back pain (LBP) on sick leave for 4 to 24 weeks. We conducted a 12-month randomized controlled trial. LBP patients were assigned to 5-day spa (2 hr/day), exercise (30 min/day) and education (45 min/day) or to usual care. The primary outcome was the percentage of patients returning to work at 1 year after randomization. Secondary outcomes were pain, disability and health-related quality of life at 1 year and number of sick leave days from 6 to 12 months. The projected recruitment was not achieved. Only 88/700 (12.6%) patients planned were enrolled: 45 in the spa therapy group and 43 in the usual care group. At 1 year, returning to work was 56.3% versus 41.9% (OR 1.69 [95% CI 0.60–4.73], p = 0.32) respectively. There was no significant difference for any of the secondary outcomes. However, our study lacked power.

## Introduction

Sick leave due to low back pain (LBP) compromises workforce productivity^1^. The costs of sick leave due to LBP are comparable to those induced by coronary heart disease, diabetes mellitus or mental disorders^2^. The chance of returning to work decreases with increasing sick leave duration^3^. Sick leave extension usually reflects the degree of perceived LBP-related symptoms and activity limitation. Other factors identified as barriers to work resumption include early negative professional experiences, fears and beliefs regarding returning to work, low self-esteem, lack of support from social security authorities and unsuitable economic arrangements^4^. Being on sick leave itself is an independent predictor of extended sick leave. Therefore, early work resumption could improve clinical outcomes in LBP patients.

The rate of persistent LBP after acute LBP usually ranges from 8% to 10%. In France, the rate of persistent LBP is 14% at 3 months^5^ and can reach 47% at 1 year in Australia^6^. Only 1 study reported outcomes in subacute LBP patients^7^. At 3 months, 40% of subacute LBP patients reported persistent LBP, 41% had a sick leave which mean duration was 30.3 (31.7) days and 82.5% had returned to work^7^. Acute and subacute periods of LBP, before 4 weeks and between 4 and 12 weeks of symptom duration, respectively, are considered a therapeutic window to prevent chronicity^8^. The main challenge at the acute and subacute periods of LBP is to detect as early as possible patients most likely to have unfavorable outcomes and to individualize the amount and type of care by the use of models to predict risk^9,10^ and of stepped approaches beginning with simple care that can be intensified if the patient does not respond^11,12^. Multidisciplinary rehabilitation is recommended for LBP patients after inappropriate response to first-line simple care^13^. At 1 year, in prospective studies conducted in France, the percentage of chronic LBP patients being at work after a multidisciplinary rehabilitation ranges between 51.4% and 85.2%^14–17^. Pain intensity while resting, perception of constant back strain when working, negative expectations for returning to work and having been to a physiotherapist could be predictors of extended sick leave in a subacute LBP population^18^. In a recent systematic review of prognostic factors for returning to work in workers with subacute and chronic LBP, Steenstra and colleagues also found that workers’ recovery expectations remained an important factor overtime^19^.

Treatments aiming to reduce sick leave duration combine multimodal approaches that include physical activities, psychobehavioral management and educational program^20–22^. Moderate-quality evidence from 8 trials suggests that multidisciplinary rehabilitation could improve the probability of being at work 1 year after an intervention (odds ratio 1.87, 95% CI 1.39 to 2.53) as compared with simple physical treatments only^21^. Inconsistently, 7 trials provided moderate-quality evidence that multidisciplinary rehabilitation do not improve the probability of being at work (odds ratio 1.04, 95% CI 0.73 to 1.47) as compared with usual care^21^. Moreover these programs are costly and their generalizability to other settings is questionable^23^.

A short but intensive multidisciplinary program represents an innovative format designed to promote returning to work. Spa centres can deliver this type of program on a community basis, which may be less disruptive than classical in- or out-patient hospital rehabilitation programs. Spa therapy, exercise therapy and educational programs combining balneotherapy, exercise therapy, physiotherapy and education are effective in relieving pain and improving function in LBP^24^ and in diminishing concomitant medication consumption in the short and long terms^25^. An educational program delivered during a 3-week spa therapy is more effective in reducing fears and deleterious beliefs than simple patient information^26^. Spa therapy is recommended by the French National Authority for Health for managing chronic LBP (strength of recommendation ranked B)^27^. However, the overall quality of trials is generally considered low^24^, and no trial has assessed returning to work.

We aimed to determine whether a 5-day multidisciplinary spa therapy is more effective than usual care in improving the percentage of returning to work at 1 year for patients with subacute and chronic LBP who are on sick leave for 4 to 24 weeks.

## Methods

### Study design

We conducted a 12-month, prospective, 2 parallel-group, multicentre, randomized controlled trial (ITILO trial) involving 5 spa centres in France. Each spa centre was affiliated with a recruiting centre located in the same region: 3 tertiary care centres (Cochin Hospital, Paris, for Thermes de Saint-Amand-les-Eaux; Clermont-Ferrand Hospital for Établissement Thermal de Royat and Nîmes Hospital for Établissement Thermal de Balaruc-les-Bains), 1 general hospital and its local network (Hôpital Thermal de Dax for Établissements Thermaux de Dax) and 1 primary care centre (general practitioner in Chambéry for Les Thermes Nationaux d’Aix-Les-Bains). To minimize the risk of performance and assessment biases that could induce an overestimation of the treatment effect, we used a modified Zelen design^28,29^, which allows for blinding of patients to the hypothesis tested. Briefly, the modified Zelen design involved 2 steps^29^: In a first step, patients were invited to participate in a cohort study to assess cLBP. The first informed consent form was about participating in this cohort. Then, patients who agreed to participate were randomized to 1 of the 2 groups. Randomization was performed on the same day as inclusion. Patients randomized to the usual care group were assessed as planned in the cohort study. Patients randomized to the spa therapy group were informed that they were randomized and that if they agreed, they would receive a 5-day spa therapy. They signed a second consent form that was about participating in spa therapy. Patients in the spa therapy group who refused the spa therapy were evaluated as specified in the first consent form they signed. Thus, patients randomized to the usual care group were not aware of an alternative therapy and therefore were less likely to experience “resentful demoralisation”, which could bias the trial results by artificially increasing the effect size of the treatment^30^. No changes in inclusion criteria or outcomes occurred after trial commencement. All the primary and secondary prespecified efficacy outcomes are reported in the present manuscript, except for the Quality-adjusted Time Without Symptoms and Toxicity (Q-TWiST) that could not be calculated because of the amount of missing data. All methods were performed in accordance with the relevant guidelines and regulations.

### Participants

Inclusion criteria were male or female, age 18 to 60 years, with subacute or chronic LBP (or LBP and radicular pain, with LBP the most painful) and sick leave for 4 to 24 weeks. Exclusion criteria were cognition or behavioral disorders disallowing assessment, inability to speak and write French and contraindication to a short spa therapy. Participants were recruited by poster advertisements in all 5 participating centres, announcements on local radio stations and local newspapers and among in- and outpatients of the rheumatology and physical medicine and rehabilitation departments of the participating centres. At Cochin centre, patients were also recruited among workers recorded as on sick leave in the electronic database of the *Assistance Publique-Hôpitaux de Paris* searched from June to November 2013 and on sick leave for LBP in the electronic database of the *Direction Régionale du Service Médical of Île-de-France* region searched on April 14, 2014 and on May 27, 2014. After invitation, individuals interested into participating contacted a management centre that confirmed the eligibility criteria, provided the patient with information about the study without mentioning spa therapy and referred the patient to a face-to-face enrolment visit. The enrolment visit was carried out by a specially trained physician from a centre independent of the spa therapy centre. Medical examination was performed during this visit.

### Intervention and control

Patients in the experimental group received a daily intensive inpatient spa therapy for 5 days at 1 of the 5 participating spa centres. The spa therapy consisted of a standardized program of spa therapy (2 hr/day in the morning) plus exercise therapy (45 min/day) plus group educational program (45 min/day in the afternoon) and receipt of the “Back Book” (5), as follows (Appendix 1):

- 2 hours of spa therapy (morning) with a trained spa technician including medical examination, pump-jet showers or whirlpool: 6 sessions (20 min at 38°), massage under water (10 min at 30°) followed by a hot shower (3 min at 38°), back and joint movements in the pool with low-back stretching (10 min at 35° of free balneotherapy followed by 15 min at 35° of global movements guided by the therapist), and mud application (10–15 min at 45–50°)
- 30 min of exercise therapy (afternoon) with a trained physiotherapist including isometric strengthening of the spinal muscles (10 min), isometric strengthening of the abdominal muscles (10 min), and isometric co-contraction of the spinal, abdominal and psoas muscles (10 min)
- 45 min of individualized educational program (afternoon) with a trained nurse or technician including:

- A first session on physical activity and rest:
  - Take-home messages about the benefits of physical activity and risks of inactivity, the meaning of an all-day activity, the reverse effects of prolonged rest
  - LBP is not a contraindication to physical activity
  - Prolonged rest over 2 days is not recommended
  - Practice of 20 min per day of physical activity is beneficial
  - A second session on physical activity using a DVD displayed by a trained therapist, then related topics are discussed between the therapist and patients
  - A third session on professional activity:
    - When sick leave is related to LBP, the longer the sick leave is, the more difficult is the returning to work and the higher is the risk of chronic LBP
    - Chronic LBP is not a contraindication to professional activity
    - Having satisfactory activity at work reduces the risk of chronic LBP
  - A fourth session on professional activity involved a DVD shown by a trained therapist, then related topics are discussed between the therapist and patients
  - A specific session on pain management with a DVD
    - Relaxation methods
    - Management of pain: self-management, self-encouragement, positive attitude, support of family or friends, being an active player in own management

- Each patient also received the “Back Book”^5,31^ that presented benefits of physical activity and the risks of inactivity, information and counselling about pain and stress management, risks of chronic pain and how to stay active.

Patients in the control group received unstandardized usual care at the discretion of their physician and the “Back Book”. Pharmacological and non-pharmacological co-interventions were allowed in both groups and were recorded in the electronic case report form (see Appendix 2).

### Outcomes

The primary outcome was the self-reported percentage of returning to work at 1 year after randomization. Secondary outcomes were: LBP recorded every 2 weeks for 1 year on a self-administered numeric rating scale for pain (NRS: 0 no pain, 100 maximal pain) and expressed by the mean area under the curve (AUC calculated using the trapezoidal rule and divided by individual follow-up duration), change from baseline in LBP-specific activity limitation assessed by the French version of the Quebec Back Pain Disability Scale (0 no limitation, 100 maximal limitation)^32,33^ and in health-related quality of life assessed by the French version of the Medical Outcomes Study 12-Item Short Form (SF-12: 0 worse health-related quality of life, 100 best health-related quality of life)^34–36^ at 1 year, Q-TWiST at 1 year, and self-reported number of sick leave days from 6 to 12 months after randomization. We have not taken into account the total number of days of sick leave from 0 to 6 months because, by definition, the intervention required patients to be available for the duration of the treatment, which would have involved sick leave. The Quebec Back Pain Disability Scale is a reliable, valid, and responsive measure of disability in back pain^37^ and the SF-12 has been included as a measure of general health status in the expanded outcome set proposed by an international group of back pain researchers, which was designed to provide more precise measurement for research purposes^38^. All the primary and secondary efficacy outcomes were collected by mail or using a secured website according to patients’ preferences. A reminding text message or email was sent.

### Safety

Adverse events (AEs) were defined as any untoward medical occurrence, which did not necessarily have a causal relationship with the clinical trial or with the experimental product. Serious adverse events (SAEs) were defined as any untoward medical occurrence that resulted in death, were life-threatening, required inpatient hospitalization or prolongation of existing hospitalization, or resulted in persistent or clinically significant disability. AEs, their attributability to the intervention and their intensity were recorded using open-ended questions during the spa therapy by the spa physician and at 3, 6 and 12 months by an investigator of each participating centre. One investigator of the main investigating centre reviewed and classified the AEs.

### Randomization and masking

An independent statistician from the *Centre d’Épidémiologie Clinique, Hôtel Dieu*, *Assistance Publique-Hôpitaux de Paris*, provided a computer-generated randomization list with permuted, variable-size blocks. The allocation ratio of assignments was 1:1. Randomization was stratified by centre. Randomization and allocation concealment were performed by the investigator who included the patient and involved use of a secured dedicated software (CleanWeb). Statisticians were blinded to the allocated group. Because of the non-pharmacological nature of the intervention, treating physicians, patients and care providers could not be blinded.

### Statistical methods

With an α risk of 0.05, a power (1-β) of 0.80, and assuming a percentage of 80% and 70% returning to work at 1 year in the spa therapy and usual care groups, respectively, we calculated that we needed 300 participants in each group. This hypothesis was formulated based on the findings of previous studies prospectively assessing the percentage of patients being at work at 1 year in French patients with chronic LBP after multidisciplinary program^14–17^. With an estimated 15% of patients lost to follow-up, we sought to include 700 patients (350 patients in each group).

For data analysis, statisticians and investigators were blinded to the treatment group allocation. Categorical variables are described with frequencies, percentages and number of missing data. Quantitative variables are described with mean (SD or 95% confidence interval [CI]) or median (interquartile range [IQR]).

Primary efficacy analysis was conducted as intent-to-treat: all randomized patients were analyzed for the primary outcome in their arm of randomization. As we performed an intent-to-treat analysis, the composition of the groups did not change after randomization. Missing data for the primary binary outcome were treated by multiple imputation by chained equation assuming the missing data to be missing at random, which allows for separating conditional distribution for each imputed variable: predictive mean matching was used for quantitative variables and logistic regression for binary variables, with m = 20 imputations. The covariates used to generate the multiple imputed data sets were age, sex, sick leave duration, LBP intensity, Quebec Back Pain Disability Scale score, centre, HADs anxiety and depression scores, employment status and educational level. Logistic regression models with fixed centre effect and fixed treatment effect were used to assess between-group difference for primary outcome at 1 year after randomization. Results were expressed as odds ratio (OR) with 95% CI and p value. Binomial regression model with an identity link was computed to derive absolute risk difference and 95% CI.

To compare between-group differences in mean changes from baseline for repeated quantitative outcomes, a constrained longitudinal data analysis proposed by Liang and Zeger was used^39^. This mixed model is constrained full-likelihood approach, whereby both the baseline and post-baseline values are modeled as dependent variables (the constrained model assumes that both the baseline and post-baseline measurements are jointly multivariate normally distributed because the baseline value is treated as part of response vector), and the true baseline means are constrained to be the same for the 2 treatment groups. Such methods based on maximum likelihood are consistent under the missing at random assumption. This model allows the inclusion of patients who are missing either the baseline or post-baseline measurements, thereby increasing efficiency. Hence, this analysis provides an adjustment for the observed baseline difference in estimating the treatment effects. Time was treated as a categorical variable so that no restriction is imposed on the trajectory of the means over time. In addition to adjusting for baseline covariate, the analysis model will also adjust for treatment, time, and interaction of time by treatment and centre. Random effect at patient level (an unstructured covariance matrix will be used to model the correlation among repeated measurements) was added. The results were expressed as differences in mean change from baseline to 1 year with 95% confidence interval.

To compare between-group differences in means for non-repeated quantitative outcomes, a general linear model was used. The analysis model adjusted for treatment and centre. The results were expressed as difference in means with 95% confidence interval.

All statistical tests were 2-sided, and *p* < 0.05 was considered statistically significant. Data were analyzed by using SAS 9.3 and 9.4 (SAS Institute Inc., Cary, NC): procedures GLIMMIX (logistic regression), MI and MIANALYZE (multiple imputation) and MIXED (constrained longitudinal data analysis model).

### Ethical consideration

The study was approved by our institutional review board (*Comité Consultatif de Protection des Personnes en Recherche Biomédicale d’Île-de-Fran*ce). All participants gave written informed consent to be in the study.

### Sources of funding and role of funders

This study was funded by the *Association Française pour la Recherche Thermale* (AFRETH 2010 program). The funding source was not involved in the design or conduct of the study or data collection, management, and analysis. It was not involved in the writing, final approval of the manuscript or decision to publish.

## Results

### Patient recruitment

**Figure 1:**
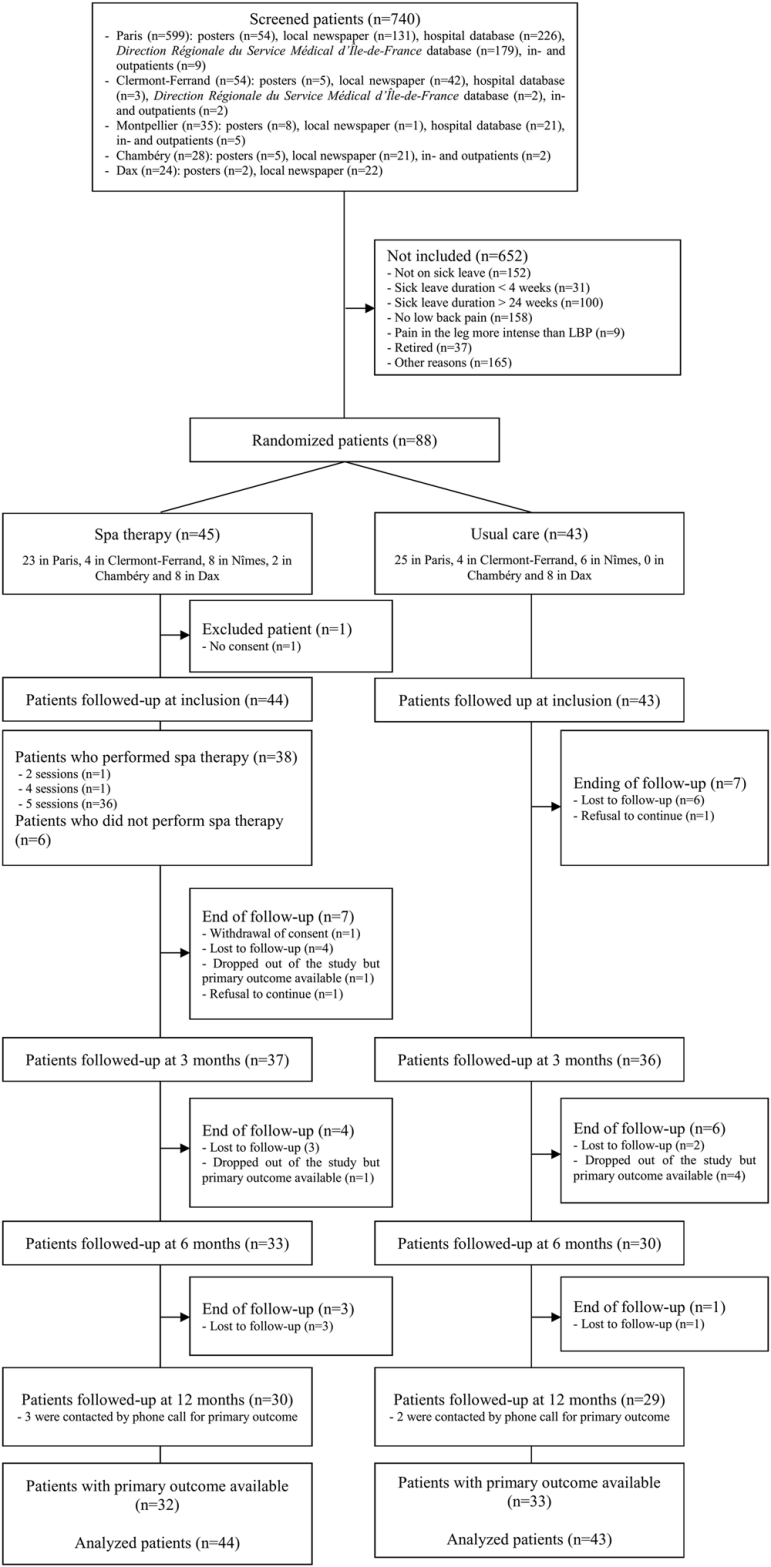
Enrollment, randomization, and follow-up.

From July 17, 2012 to July 17, 2014, 740 LBP patients were screened (599, 54, 35, 28 and 24 in Paris, Clermont-Ferrand, Montpellier, Chambéry and Dax, respectively). Overall, 88 patients met inclusion criteria and were randomly assigned to the spa therapy group (n = 45) or usual care group (n = 43). Among the 45 patients allocated to the spa therapy group, 1 patient was excluded from the primary efficacy analysis because informed consent could not be obtained and 6 patients did not receive spa therapy but were analyzed for the primary outcome in their arm of randomization (Fig. 1). The median age was 47.0 years (IQR: 37.0–53.0) and the male to female ratio was 2:3. Median symptom and sick leave durations were 4.9 (IQR: 3.3–6.4) and 3.7 (IQR: 1.8–4.9) months, respectively. The mean (SD) time elapsed between randomization and the first spa therapy day in the spa therapy group was 24.7 days (16.8) (Table 1).

**Table 1:** Baseline demographics and low back pain (LBP) characteristics of patients with spa therapy, exercise therapy and educational program and usual care.

	Spa therapy (n = 44)	Usual care (n = 43)	All patients (n = 87)
Age (years), median (IQR)	47.0 (37.5;52.5)	47.0 (37.0;53.0)	47.0 (37.0;53.0)
Female, n (%)	26/44 (59.1)	25/43 (58.1)	51/87 (58.6)
Educational level, n (%)			
High school or less	29/44 (66.0)	34/43 (79.1)	63/87 (72.6)
Higher education	15/44 (34.1)	9/43 (20.9)	24/87 (27.6)
Employment status			
Sick leave duration (months), median (IQR)	3.5 (2.1;4.5)	3.7 (1.7;4.9)	3.7 (1.8;4.9)
Last occupation, n (%)			
Artisan, trader, manager	0/44 (0.0)	1/44 (2.3)	1/87 (1.1)
Senior executive, intellectual profession	3/44 (6.8)	2/43 (4.7)	5/87 (5.7)
Intermediate occupation	2/44 (4.5)	2/43 (4.7)	4/87 (4.6)
Employee	37/44 (84.1)	34/43 (79.1)	71/87 (81.6)
Worker	2/44 (4.5)	4/43 (9.3)	6/87 (6.9)
LBP duration (months), median (IQR)	4.6 (3.0;6.1)	5.5 (3.3;7.1)^a^	4.9 (3.3;6.4)
LBP intensity in the last 48 hr (NRS, 0–100), mean (SD)	58.0 (19.9)	58.7 (21.7)	58.3 (20.7)
Radicular pain intensity in the previous 48 hr (NRS, 0–100), mean (SD)	32.5 (24.6)	31.5 (27.8)	32.0 (26.1)
Main imaging finding, n (%)			
Lumbar spinal stenosis	1/44 (2.3)	0/43 (0)	1/87 (1.1)
Facet joint osteoarthritis	1/44 (2.3)	1/43 (2.3)	2/87 (2.3)
Spondylolisthesis	0/44 (0.0)	1/43 (2.3)	1/87 (1.1)
Scoliosis	0/44 (0.0)	1/43 (2.3)	1/87 (1.1)
Degenerative disc disease	25/44 (56.8)	25/43 (58.1)	50/87 (57.5)
Discoradicular conflict	11/44 (25.0)	10/43 (23.3)	21/87 (24.1)
Active discopathy	3/44 (6.8)	3/43 (7.0)	6/87 (6.9)
Scheuermann’s disease	0/44 (0)	1/43 (2.3)	1/87 (1.1)
Unspecified	3/44 (6.8)	1/43 (2.3)	4/87 (4.6)
Quebec Back Pain Disability Scale (0–100), mean (SD)	49.3 (17.0)^a^	49.3 (14.9)	49.3 (15.9)
SF-12 PCS (0–100), mean (SD)	30.4 (5.0)^a^	31.2 (6.2)	30.8 (5.6)
SF-12 MCS (0–100), mean (SD)	36.9 (9.7)^a^	35.1 (6.9)	36.0 (8.4)
HADs anxiety score (0–21), mean (SD)	11.5 (4.0)^a^	11.2 (4.0)	11.4 (4.0)
HADs depression score (0–21), mean (SD)	8.8 (3.9)^b^	7.7 (3.2)	8.2 (3.6)
FABQ physical activity score (0–24), median (IQR)	16.0 (12.0;20.0)^c^	17.0 (12.0;22.0)	17.0 (12.0;20.0)
FABQ work score (0–42), median (IQR)	35.5 (29.0;39.0)^a^	35.0 (29.0;39.0)	35.0 (29.0;39.0)
Previous treatments, n (%)			
Analgesics	42/44 (95.5)	42/43 (97.7)	84/87 (96.6)
NSAIDs	41/44 (93.2)	39/43 (90.7)	80/87 (92)
Muscle relaxants	28/44 (63.6)	34/43 (79.1)	62/87 (71.3)
Anxiolytics	12/44 (27,3)	11/43 (25.6)	23/87 (26.4)
Antidepressants	10/44 (22.7)	11/43 (25.6)	21/87 (24.1)
Anticonvulsants	4/44 (9.1)	8/43 (18.6)	12/87 (13.8)
Lumbar spinal injections	20/44 (45.5)	25/43 (58.1)	45/87 (51.7)
Lumbar brace	33/44 (75)	36/43 (83.7)	69/87 (79.3)
Physiotherapy	35/44 (79.5)	34/43 (79.1)	69/87 (79.3)
Rehabilitation program	6/44 (13.6)	6/43 (14)	12/87 (13.8)
Alternative medicine	17/44 (38.6)	15/43 (34.9)	32/87 (36.8)
Time between randomization and spa therapy (days), mean (SD)	24.7 (16.8)	—	—

FABQ: Fear And Beliefs Questionnaire; HADs: Hospital Anxiety Depression scale; IQR: interquartile range; MCS: mental component subscale; NRS: numeric rating scale; NSAIDs: non-steroidal anti-inflammatory drugs; PCS: physical component subscale; SF-12: Medical Outcome Survey 12-Item Short Form.

^a^n = 42, ^b^n = 41, ^c^n = 40.

### Primary outcome

At 1 year, there was no statistically significant difference between spa therapy and usual care groups for the primary outcome: the percentage of returning to work was 56.3% versus 41.9%, respectively (odds ratio after multiple imputation: 1.69 [95% CI 0.60 to 4.73], p = 0.32, absolute difference 12.6% [95% CI −12.0% to 37.2%]) (Table 2).

**Table 2:** Percentage of returning to work at 1 year (primary outcome).

	Spa therapy n = 44	Usual care n = 43	Absolute difference (95% CI)^†^	Odds ratio (95% CI)^††^	p-value
Without imputation					
Percentage of returning to work at 1 year, n (%)	18/32 (56.3)	14/33 (42.4)	13.1 (−11.6;37.7)	1.72 (0.62;4.76)	
After multiple imputation (mean of 20 imputations)					
Percentage of returning to work at 1 year, %	56.3	41.9	12.6 (−12.0;37.2)	1.69 (0.60;4.73)	0.32

CI: confidence interval.

^†^Spa therapy group *minus* usual care group, difference of percentages adjusted on centre.

^††^Spa therapy group *versus* usual care group, odds ratio adjusted on centre.

### Secondary outcomes

There was no statistically significant difference between the 2 groups for any of the secondary outcomes (Table 3). Q-TWiST could not be calculated because of the amount of missing data.

**Table 3:** Secondary outcomes.

Outcome	Spa therapy n = 44	Usual care n = 43	Adjusted difference^†^ (95% CI)	p-value
Mean AUC for LBP (NRS, 0–100), mean (95% CI)^*^	39.9 (31.5;48.4)	43.5 (34.3;52.6)	−3.5 (−11.7;4.0)	0.36
Change from baseline to 1 year in Quebec Back Pain Disability Scale (0–100)^a^, mean (95% CI)^**^	−12.2 (−18.9;−5.6)	−5.2 (−11.9;1.5)	−7.1 (−16.3;2.2)	0.13
Change from baseline to 1 year in SF-12 PCS (0–100)^a^, mean (95% CI)^**^	5.2 (2.3;8.2)	5.5 (2.5;8.5)	−0.3 (−4.5;3.9)	0.89
Change from baseline to 1 year in SF-12 MCS (0–100)^a^, mean (95% CI)^**^	8.1 (3.4;−12.7)	5.1 (0.4;9.8)	3.0 (−3.5;9.4)	0.36
No of sick leave days between 6 to 12 months after randomization date^b^, mean (95% CI)^*^	43.7 (0.0;95.6)^***^	40.1 (0.0;95.6)^***^	3.6 (−47.4;54.6)	0.89

^†^Spa therapy *minus* usual care.

^*^Values adjusted on centre.

^**^Values adjusted on baseline value and centre.

^***^The lower limit of the confidence interval was truncated at zero.

AUC: area under the curve; LBP: low back pain; MCS: mental component score; NRS: numeric rating scale; PCS: physical component score; SF-12: 12-item short-form general health survey.

Q-TWIST could not be calculated because of the amount of missing data.

^a^n = 29 in the spa therapy group and n = 28 in the usual care group at 1 year, ^b^n = 32 in the spa therapy group and n = 33 in the usual care group at 1 year.

### Compliance

Overall, 36/44 (81.8%) patients attended the 5 days of the spa therapy (Fig. 1).

### Safety

A total of 47 AEs were recorded: 22/47 (46.8%) in the spa therapy group versus 25/47 (53.2%) in the usual care group. Overall, 13 AEs were considered severe (6 in the spa therapy group and 7 in the usual care group): 6 patients reported increased LBP (2 in the spa therapy group and 6 in the usual care group) and 7 patients reported hospitalisations or care for unrelated disorder to LBP (4 in the spa therapy group and 3 in the usual care group). None was attributed to the intervention (see Appendix 3).

## Discussion

In this 12-month, prospective, 2 parallel-group, multicentre, randomized controlled trial, our study sample size was too small (12.6% of calculated sample size) to allow providing definitive conclusion on the effect of a 5-day spa therapy.

Even though LBP is the most prevalent musculoskeletal condition among the French population of working age and the most frequent cause of sick leave^40^, the main limitation of our study, was the insufficient number of patients fulfilling the inclusion criteria despite a large screening. Under-recruitment to randomized controlled trials has long been a problem^41–44^, with various strategies developed to overcome barriers to recruitment^45–47^. Before starting our study, we planned a triple approach to optimize patient recruitment: (1) locoregional recruitment, with each spa centre affiliated with a recruiting centre of the same region. This approach was efficacious in the Thermarthrose randomized controlled trial of 400 patients with knee osteoarthritis, with patients living in an area located near the spa centre^48^; (2) recruitment via general practitioners (GPs) and specialist primary care networks^49^; and (3) recruitment by advertising in local media and among individuals working in the participating tertiary care centres. In addition, at the Cochin centre, specific databases of patients on sick leave were searched and patients were invited by mail to participate in the study. Despite these measures, we faced several issues including low rate of recruitment from primary care, limited access to reliable database of patients with acute or subacute LBP in sick leave for 4 to 24 weeks and limited possibility to promote the study due to its Zelen design.

A substantial number of barriers contribute to the low implementation of clinical research in general practice. For example, a recent study found that many issues were related to the German market-based healthcare and academic systems and traditions^50^. As in France, in Germany, most GPs work in a market-based, competitive setting of small private practices, with a high case load, have no protected time or funding for research, and have mostly no research training or experience^50^. Conversely, in the United Kingdom, primary care clinical trials in practice-based research networks are considered a priority within national funding programs^51^, and substantial efforts have been made to facilitate trials in general practice settings, including training and accreditation of “research ready” practices. In our study, the low rate of recruitment from primary care might be explained by (1) lack of a well-established and active network of GPs and specialists involved in practice-based research in France, (2) lack of institutional financial support for their participation in clinical studies and (3) lack of a full implementation of academic general practice.

Another problem was access to an updated database of patients with subacute or chronic LBP on sick leave for 4 to 24 weeks. Access to health care system databases for research purposes in France is restricted by the *Commission Nationale de l’Informatique et des Libertés*, which usually induces long delays between authorization of requests for searches and actually obtaining the lists of patients. Contrary to some other countries, such as The Netherlands, the United Kingdom or the United States, where practice-based research networks provide continuous high-quality observational data dedicated to research and serve as a platforms for clinical trials^50^, few databases in France are designed for research. Therefore, medical conditions are usually poorly described, difficult to extract and sometimes outdated. For example, at the Cochin centre, the search of *Assistance Publique-Hôpitaux de Paris* and *Direction Régionale du Service Médical d’Île-de-France* databases yielded 10,535 responses for the specified periods (8,240 patients recorded as on sick leave and 2,295 on sick leave for LBP). After the exclusion of duplicate files and sick leave durations shorter than 4 weeks and longer than 24 weeks, 4,916 patients could eventually contacted by mail. The response rate was 12.2% (n = 599) and 5.2% (n = 255), respectively. Some patients might have been reluctant to participate in an unsolicited clinical trial.

Communication about the study was limited by the modified Zelen design. The conventional process of randomized trials is to fully informed participants of the treatments being compared and to require consent before randomizing participants. In the context of a trial comparing an intervention to usual care, where blinding is impossible, this design raises the risk of resentful demoralization and detection bias. The modified Zelen design allows informing only participants in the intervention group after randomization. This design nevertheless raises some issues when the number of participants refusing the experimental treatment is high as it will reduce the study power^52,53^. This type of design might also have dissuaded them from accepting enrollment because no specific intervention seemed to be proposed at first.

As reviewed by Poiraudeau and colleagues, most published studies report favorable returning to work rates at 1 and 2 years (from 65 to 90%) after multidisciplinary rehabilitation. However these rates vary across countries with different work compensation systems^15^. Longitudinal studies in France have shown homogenous results in returning to work rates at 1 year ranging from 51.4% (54/105 patients)^14^ to 85.2% (52/61 patients)^17^. In one of the first French open prospective study assessing the efficacy of multidisciplinary program for returning to work, Poiraudeau and colleagues found in 35 patients with disabling chronic LBP that a 5-week intensive inpatient program which main components were specific exercises to increase trunk, lower and upper limb muscles flexibility and strength, training in functional tasks, education and work endurance was associated to a percentage of returning to work at 1 year of 66.0% (24/35 patients)^16^. These results have further been confirmed by 3 independent French groups in 2 open prospective^14,17^ and 1 retrospective studies^54^ with consistent returning to work rates at 1 year. However, in the only randomized controlled trial conducted in France comparing the efficacy on several outcomes at 1 year of multidisciplinary rehabilitation to outpatient active physiotherapy (1 hour 3 times a week, during 5 weeks), Roche-Leboucher and colleagues found no between-group difference in the rate of returning to work at 1 year which was high in both groups (93.8% vs 85.4%)^55^, suggesting that less intensive rehabilitation programs could also improve professional outcomes. With this regard, our findings that returning to work rate at 1 year was 56.3% in the spa therapy group (absolute between-group difference after multiple imputation = 12.6%, 95% CI from −12.0 to 37.2%) are consistent with the rates previously reported with more intensive multidisciplinary rehabilitation. Cost-effectiveness studies may help in establishing a hierarchy among multidisciplinary approaches.

Strengths and limitations. Our inclusion and exclusion criteria and screening methods may have represented a limitation to the external validity of our findings. However, our inclusion strategy was consistent with existing knowledge in the literature in similar setting in France for knee osteoarthritis^48^ and LBP^26^. Furthermore, our results may be valid for French patients with chronic LBP and who are on sick leave. However, generalizability and acceptability of this type of program in other settings need to be further assessed. Finally, we did not record whether participants in the control group received spa therapy. However, considering the difficulties of having access to spa therapy outside of the trial (in France it implies a specific prescription and agreement to be reimbursed), we believe the risk of contamination was probably low.

## Conclusions

Our study lacked power. However, it might be considered an original proof-of-concept pilot study. We believe that our preliminary findings are promising and support further large-scaled studies assessing this type of strategy with a design specifically accounting for the limitations we encountered with recruitment. Such a study might be performed in countries where primary care networks participating in clinical research are more well-established and databases are more accessible than in France.

### Availability of data and materials

Full original protocol, final protocol, summary of changes, and full original statistical analysis plan, final statistical analysis plan, summary of changes can be accessed upon request for academic researchers by contacting Assoc Prof Christelle Nguyen (christelle.nguyen2@aphp.fr).

## Electronic supplementary material

CONSORT Checklist

Full protocol
