# Supplementary material for: Intensive spa and exercise therapy program for returning to work for low back pain patients: a randomized controlled trial
Source: Sci Rep. 2017 Dec 20;7:17956. doi: 10.1038/s41598-017-18311-z (PMC5738382; doi:10.1038/s41598-017-18311-z)
Supplement: Supplementary file 2 — Full protocol [file 41598_2017_18311_MOESM2_ESM.doc]

**Effectiveness of a Short, Intensive and Standardised Spa Therapy for Subacute and Chronic Low Back Pain on Return to Work for Patients in Sick Leave From 4 to 24 Weeks Duration (ITILO)**

**VERSION no. 1.0 of 26 December 2011**

**Sponsor:** Association Française pour la Recherche Thermale

1, rue Cels

75014 Paris

Tel. (+33) 01 53 91 05 77 / Fax: (+33) 01 43 21 01 80

**Principal Investigator:** Prof. Serge Poiraudeau

Department of Physical Medicine and Rehabilitation of the Musculoskeletal System and Spinal Disorders

Hôpital Cochin,

27 rue du Faubourg St Jacques

75679 Paris Cedex 14

Tel. (+33) 01 58 41 25 49 / Fax: (+33) 01 58 41 25 45

**Methodology and statistical analysis:** Dr Isabelle Boutron, Prof. Philippe Ravaud,

Clinical Epidemiology Centre

Hôpital Hôtel Dieu

1 place du Parvis Notre Dame

75004 Paris

**Clinical Research Unit**: URC CIC Cochin Necker

Site Tarnier

89 rue d’Assas

75006 Paris

Tel. (+33) 01 58 41 28 84 / Fax: (+33) 01 58 41 11 83

**Biomedical research PROTOCOL SIGNATURE page**

**for the Principal Investigator and the sponsor’s representative**

Biomedical research no. *code:* ***ITILO***

*Title:* “**Effectiveness of a Short, Intensive and Standardised Spa Therapy for Subacute and Chronic Low Back Pain on Return to Work for Patients in Sick Leave From 4 to 24 Weeks Duration”**

Version no. 1.0 of: 26/12/2011

| Principal Investigator: |  |
| --- | --- |
| Prof. Serge Poiraudeau |  |
| Department of Physical Medicine and Rehabilitation of the Musculoskeletal System and Spinal Disorders  Hôpital Cochin, | *Date 26/12/2011* |
| 27 rue du Faubourg St Jacques  75679 PARIS Cedex 14 | Signature:  [Signature] |
|  | |
|  | |
|  | |
|  |  |
| **Sponsor:** |  |
|  |  |
|  | *Date: ……/………/………..* |
|  | Signature: |
|  |  |
|  |  |

Please note: This version corresponds to the text from the protocol and annexes sent to the Ethics Committee (CPP) for its opinion and to the competent authority for authorisation.

*If another version is prepared at a later stage following any amendments, the signature process must be repeated to ensure the active protocol versions are always up to date.*

# TABLE OF CONTENTS

[*TABLE OF CONTENTS 3*](#__RefHeading___Toc469080350)

[SUMMARY 5](#__RefHeading___Toc469080351)

[GENERAL OUTLINE OF THE STUDY 8](#__RefHeading___Toc469080352)

[I - INTRODUCTION 10](#__RefHeading___Toc469080353)

[I - 1 - DATA FROM THE LITERATURE 10](#__RefHeading___Toc469080354)

[I - 2 - JUSTIFICATION OF THE RESEARCH 11](#__RefHeading___Toc469080355)

[I - 3 - EXPECTED RESULTS AND PROSPECTS 11](#__RefHeading___Toc469080356)

[I - 4 - Study feasibility 12](#__RefHeading___Toc469080357)

[I - 5 - Expected benefits and risks 12](#__RefHeading___Toc469080358)

[II – STUDY OBJECTIVE 13](#__RefHeading___Toc469080359)

[II - 1 - PRIMARY OBJECTIVE 13](#__RefHeading___Toc469080360)

[II - 2 - SECONDARY OBJECTIVES 13](#__RefHeading___Toc469080361)

[III – METHODOLOGY 13](#__RefHeading___Toc469080362)

[III - 1 - STUDY TYPE 13](#__RefHeading___Toc469080363)

[III - 2 - STUDY DURATION 13](#__RefHeading___Toc469080364)

[III - 3 – RANDOMISATION ARM ASSIGNMENT 13](#__RefHeading___Toc469080365)

[III - 4 – EXPERIMENTAL DESIGN 14](#__RefHeading___Toc469080366)

[IV – STUDY POPULATION 16](#__RefHeading___Toc469080367)

[IV - 1 - INCLUSION CRITERIA 16](#__RefHeading___Toc469080368)

[IV - 2 - EXCLUSION CRITERIA 16](#__RefHeading___Toc469080369)

[IV - 3 - REQUIRED NUMBER OF SUBJECTS 16](#__RefHeading___Toc469080370)

[IV - 4 - RECRUITMENT METHOD 16](#__RefHeading___Toc469080371)

[V – STUDY TREATMENT 17](#__RefHeading___Toc469080372)

[V - 1 – Intensive spa THERAPY 17](#__RefHeading___Toc469080373)

[V - 2 – ASSOCIATED TREATMENTS 19](#__RefHeading___Toc469080374)

[VI – ENDPOINTS 19](#__RefHeading___Toc469080375)

[VI - 1 - PRIMARY ENDPOINT 19](#__RefHeading___Toc469080376)

[VI - 2 - SECONDARY ENDPOINTS 19](#__RefHeading___Toc469080377)

[VII – PATIENT STUDY PLAN 19](#__RefHeading___Toc469080378)

[VII - 1 – D0 - ENROLMENT VISIT 19](#__RefHeading___Toc469080379)

[VII - 2 - INTERVENTIONAL PROTOCOL 21](#__RefHeading___Toc469080380)

[VII - 3 - FOLLOW-UP VISITS after the intervention 21](#__RefHeading___Toc469080381)

[VII - 4 - EXITING THE TRIAL AND EARLY WITHDRAWAL 22](#__RefHeading___Toc469080382)

[VII - 5 - STUDY TERMINATION 22](#__RefHeading___Toc469080383)

[VIII – QUALITY CONTROL AND ASSURANCE 22](#__RefHeading___Toc469080384)

[VIII - 1 - MONITORING PROCEDURES 23](#__RefHeading___Toc469080385)

[VIII - 2 – TRANSCRIPTION OF INFORMATION INTO THE CASE REPORT FORM 23](#__RefHeading___Toc469080386)

[IX – STATISTICAL DATA MANAGEMENT 25](#__RefHeading___Toc469080387)

[IX -1- PLANNED STATISTICAL METHODS 25](#__RefHeading___Toc469080388)

[IX -1- 1- Descriptive statistics 25](#__RefHeading___Toc469080389)

[IX -1- 2- Baseline criteria 25](#__RefHeading___Toc469080390)

[IX -1- 3- Early withdrawals from the study 26](#__RefHeading___Toc469080391)

[IX -1- 4- Compliance and duration of the study 26](#__RefHeading___Toc469080392)

[IX -1- 5- Primary endpoint analysis 26](#__RefHeading___Toc469080393)

[IX -1- 6- Secondary analyses 26](#__RefHeading___Toc469080394)

[IX -1- 7- Tolerance 27](#__RefHeading___Toc469080395)

[IX -2 - STATISTICAL CRITERIA FOR TERMINATING THE RESEARCH 27](#__RefHeading___Toc469080396)

[IX -3 - METHOD FOR TAKING INTO ACCOUNT MISSING, UNUSED AND INVALID DATA 27](#__RefHeading___Toc469080397)

[IX - 4- CHOICE OF INDIVIDUALS TO BE INCLUDED IN THE ANALYSES 27](#__RefHeading___Toc469080398)

[IX - 5 - DESCRIPTION OF PROTOCOL DEVIATIONS 27](#__RefHeading___Toc469080399)

[X – SAFETY ASSESSMENT 28](#__RefHeading___Toc469080400)

[X - 1 - DESCRIPTION OF THE SAFETY ASSESSMENT PARAMETERS 28](#__RefHeading___Toc469080401)

[X - 2 - Adverse events 28](#__RefHeading___Toc469080402)

[X - 3 - SERIOUS ADVERSE EVENT MANAGEMENT PROCEDURE 29](#__RefHeading___Toc469080403)

[X - 4 - STEERING COMMITTEE 29](#__RefHeading___Toc469080404)

[XI – RIGHT TO ACCESS THE INFORMATION AND SOURCE DOCUMENTS 29](#__RefHeading___Toc469080405)

[XII – LEGAL AND ETHICAL ASPECTS 30](#__RefHeading___Toc469080406)

[XII - 1 - REQUEST FOR AUTHORISATION FROM THE ANSM 30](#__RefHeading___Toc469080407)

[XII - 2 - REQUEST FOR ETHICS COMMITTEE OPINION 30](#__RefHeading___Toc469080408)

[XII - 3 - AMENDMENTS 30](#__RefHeading___Toc469080409)

[XII - 4 - CNIL DECLARATION 31](#__RefHeading___Toc469080410)

[XII - 5 - Information sheet and consent form 31](#__RefHeading___Toc469080411)

[XII - 6 - FINAL REPORT ON THE RESEARCH 31](#__RefHeading___Toc469080412)

[XIII – DATA PROCESSING AND STORAGE OF DOCUMENTS AND DATA RELATING TO THE RESEARCH STUDY 32](#__RefHeading___Toc469080413)

[XIV – INSURANCE AND SCIENTIFIC COMMITMENT 32](#__RefHeading___Toc469080414)

[XIV - 1 - INSURANCE 32](#__RefHeading___Toc469080415)

[XIV - 2 - SCIENTIFIC COMMITMENT 33](#__RefHeading___Toc469080416)

[XV – RULES REGARDING PUBLICATION 33](#__RefHeading___Toc469080417)

[XVI – LIST OF ANNEXES 33](#__RefHeading___Toc469080418)

[ANNEX I – BIBLIOGRAPHY REFERENCES 34](#__RefHeading___Toc469080419)

[ANNEX II – INVESTIGATORS AND ASSOCIATE TEAMS 36](#__RefHeading___Toc469080420)

[ANNEX III – Standardised spa therapy protocol 37](#__RefHeading___Toc469080421)

# SUMMARY

| **Title** | Effectiveness of a Short, Intensive and Standardised Spa Therapy for Subacute and Chronic Low Back Pain on Return to Work for Patients in Sick Leave From 4 to 24 Weeks Duration |
| --- | --- |
| **Principal Investigator** | Prof. Serge Poiraudeau (Hôpital Cochin) |
| **Recruiting sites** | Hôpital Cochin AP-HP Paris, Hôpital Nord CHU Clermont-Ferrand, Hôpital Lapeyronie CHU Montpellier, CHU Nancy, Etablissements thermaux d’Aix les Bains et Dax |
| **Spa centres** | Saint Amand les eaux, Royat, Balaruc les Bains, Amnéville les Thermes, Aix les bains et Dax. |
| **Study duration** | 36 months (24-month enrolment period) |
| **Patient follow-up** | 12 months |
| **Study aim** | Primary objective:  To evaluate the efficacy of a short, standardised spa therapy intervention on the return-to-work rates at 1 year for patients on sick leave due to lower back pain from 4 to 24 weeks’ duration.  Secondary objectives:  To evaluate the efficacy of a short, standardised spa therapy intervention on the pain, function, quality of life and number of days of sick leave at 1 year for patients on sick leave due to lower back pain from 4 to 24 weeks’ duration. |
| **Methodology** | A multi-centre, comparative, randomised, pragmatic trial using a modified ZELEN method. |
| **Number of sites** | 6 recruiting sites and 6 spa centres |
| **Number of patients** | 700, of whom:  - 350 in the intervention arm (standardised spa treatment)  - and 350 in the control arm (standard treatment) |
| **Selection criteria** | Inclusion criteria:   - Men or women aged from 18 to 60 years (inclusive); - Isolated lower back pain or back and radicular pain after classification of the disease (with the lower back numeric pain scale score being higher than the radicular numeric pain scale score); - Patients on sick leave due to common lower back pain from 4 to 24 weeks’ duration and for those in which an extended period of sick leave is expected; - Medical examination carried out in advance, the results of which will be communicated to the patient; - Patients having provided their written informed consent to take part in the study; - Patients affiliated with a social security scheme (beneficiary or entitled).   Exclusion criteria:   - Cognitive or behavioural difficulties making the assessment impossible; - Difficulties in comprehension and communication in the French language, making the assessment impossible; - Contraindication to undergo a short spa therapy programme. |
| **Study treatment** | Short, standardised spa therapy (5 days). |
| **Study progress** | One enrolment visit, followed by the intervention protocol for patients in the “standardised spa therapy” arm, then follow-up by mail at Month 3, 6 and 12.  D0 - Enrolment visit and randomisation   - Medical examination - Verification of eligibility criteria - Proposal of cohort study and collection of signed informed consent form - Enrolment - Patient contact information (name and surname, email, phone number) - Demographic information (age, sex) - Socioprofessional information (level of studies and profession) - Date sick leave started - Clinical information regarding lower back pain - Previous and ongoing treatment for lower back pain - Numeric pain scale - Quebec questionnaire - HAD questionnaire - Quality of Life, assessed with: MOS SF12 - FABQ questionnaire - CSQ questionnaire - Randomisation - Proposal of therapy for patients randomised into the intervention group and collection of signed informed consent form   Intervention protocol for patients randomised into the “spa therapy” arm   - Duration: five days, consisting of spa therapy sessions (exercises and hydrotherapy) and group therapy education   Assessments at Months 3, 6 and 12   - By post or email, together with a phone call if no response is received - Professional status/Return to work - Sick leave periods since the previous visit - Numeric pain scale - Lower back pain progress - Treatment since the previous visit - Adverse events collected - Quebec questionnaire - HAD questionnaire - MOS SF-12 questionnaire - FABQ questionnaire (only at the Month 12 assessment) - CSQ questionnaire (only at the Month 12 assessment) - Treatment satisfaction at 12 months   Pain assessment every fortnight  Numeric pain scale via telephone (IVR system) |
| **Primary and secondary**  **endpoints** | Primary endpoint  The primary endpoint will be the return-to-work rate at 1 year.  Secondary endpoints  The secondary endpoints will be as follows:   - Area under the curve of pain over the past 48 hours, assessed every fortnight by a numeric scale from 0 to 100 at 1 year - Functional assessment at 1 year (Quebec scale) - Quality of Life assessment at 1 year (MOS SF-12 questionnaire) - TWIST at 1 year (pain) - Number of days of sick leave between 6 months and 1 year after enrolment |
| **Statistical analysis** | An intention-to-treat analysis will be carried out with the data. The return-to-work rate percentage will be compared using the Chi-squared test. Area under the curve of pain and the TWIST will be compared using the Mann–Whitney U test. The other quantitative secondary endpoints will be compared using a linear mixed effects regression model.  The statistical analysis will be carried out at the Prof. Ravaud Clinical Epidemiology Centre - Hôtel Dieu, under the responsibility of Dr I. Boutron and G. Baron. SAS software will be used. |

# GENERAL OUTLINE OF THE STUDY

Management centre: elegibility criteria

**Enrolment visit**

Collection of ICF1 for participation in a cohort study lasting 1 year

**Randomisation**

Intervention arm

Standardised spa therapy

Control arm

Standard treatment

Collection of ICF2 for participation

Assessment at D0

**Local recruitment (local press**

**posters in pharmacies, doctors’ waiting**

**rooms) and at medical visits**

Assessment at D0

Intervention (spa therapy, group therapeutic education and back guide)

Follow-up at 3 months, 6 months and 1 year by post or email

+ numeric pain scale every fortnight by telephone

|  | **Enrolment visit + randomisation** | **Intervention**  **Visit** | **Visit 1**  **Mail/**  **phone** | **Visit 2**  **Mail/**  **phone** | **Visit 3**  **Mail/**  **phone** |
| --- | --- | --- | --- | --- | --- |
|  | D0 | M1-M3  Maximum after intervention | M3 | M6 | M12 |
| Inclusion and exclusion criteria | + |  |  |  |  |
| **Information and consent for the cohort study** | + |  |  |  |  |
| **Randomisation** | + |  |  |  |  |
| **Information and consent to participate in the therapy for patients randomised into the experimental arm** | + |  |  |  |  |
| **Patient contact information** | + |  |  |  |  |
| **Medical examination** | + |  |  |  |  |
| **Back guide and patient diary issued** | + |  |  |  |  |
| ASSESSMENT: | | | | | |
| - Socioprofessional information | + |  |  |  |  |
| - Clinical information regarding lower back pain | + |  | + | + | + |
| - Number of days of sick leave | + |  | + | + | + |
| - Return to work |  |  | + | + | + |
| - Number of periods of sick leave |  |  | + | + | + |
| - Treatments | + |  | + | + | + |
| - Numeric pain scale | + |  | + | + | + |
| - Quebec | + |  | + | + | + |
| - HAD | + |  | + | + | + |
| - MOS SF-12 | + |  | + | + | + |
| - FABQ | + |  |  |  | + |
| - CSQ | + |  |  |  | + |
| - Treatment satisfaction scale |  |  |  |  | + |
| **Adverse Events** |  | + | + | + | + |
| **INTERVENTION: Intensive spa therapy** |  | + |  |  |  |

# I - INTRODUCTION

## I - 1 - DATA FROM THE LITERATURE

Disability and sick leave related to lower back pain represent a major public health problem in developed countries (Andersson GB, Lancet 1999) and their costs to the healthcare system are comparable to those of heart disease, diabetes and depression (Druss BG, Am J Psychiatr 2000). The longer the sick leave lasts, the less likely it is that the patient will return to work (Waddell G, Occupational Medicine 2001). Extended periods of sick leave can reflect the level of complaints and functional incapacity, but being on sick leave could be an independent risk factor of extended sick leave. Factors such as professional experiences considered to be negative, low level of confidence in capacity to return to work and a low level of self-esteem have been identified as barriers for returning to work in this clinical situation (Magnuson L, Disab Rehabil 2007). Aside from the financial benefits, returning to work may also be a therapeutic improvement factor for lower back pain.

The rate of transition from acute lower back pain to chronic form is currently the subject of debate, as recent studies appear to contradict the previously stated rates of 8% to 10% with persistent pain as reported at 1 year in 47% in an Australian study (Menezes L, BMJ 2009) and 14% at 3 months in a French study (Coudeyre E, PloS ONE 2007). Subacute lower back pain (lasting between 4 and 12 weeks), however, is recognised as a preferred therapeutic target for preventing the transition to chronic form and the consequences this would have in terms of disability and treatment costs (Savigny P, BMJ 2009). Various interventions, which are often multidisciplinary, have shown an interest in reducing the intensity of the symptoms in this situation, but a significant number of patients have delayed symptoms and are on sick leave (Anema JR, Spine 2007).

Only one study has reported the progress of patients suffering from subacute lower back pain in France (Poiraudeau S, Rheumatol 2006). Around half of the patients were still symptomatic at 3 months, 41% of them had resorted to sick leave lasting an average of 30 days, and 20% had extended periods of sick leave. The non-return-to-work factors in subacute lower back pain have not been widely studied. A recent Norwegian study reported that the pain intensity at rest and at work and negative expectations related to return to work were non-return factors (Reme SE, BMC Musculoskeletal Dis 2009).

The most effective treatments for chronic lower back pain in terms of return to work are multidisciplinary programmes that combine physical activities with psycho-behavioural treatment (Schonstein E, Cochrane Database of Systematic Reviews 2003). However, most programmes proposed are intense (3 to 6 weeks) and are proposed at a late stage in cases of chronic lower back pain on extended sick leave (> 6 months). Some studies suggest, however, that less intense treatment proposed at an earlier stage could lead to a higher return-to-work rate than with standard care (Karjalaïnen JA, Cochrane Database of Systematic Reviews 2003, Anema JR, Spine 2007). There have been no therapeutic trials conducted on the subject in France and it is a known fact that the treatment and social protection system in this area has an influence on the return-to-work rate (Hanson, Spine 2000), which makes it difficult to generalise with regard to the results obtained in one health system or another.

## I - 2 - JUSTIFICATION OF THE RESEARCH

This is a multi-centre, randomised, pragmatic, intervention study comparing a short (5-day) standardised spa therapy combined with intensive rehabilitation and education in a spa therapy centre to the standard treatment for patients on sick leave from 4 to 24 weeks’ duration due to common lower back pain.

Why have we proposed such a study?

Patients on sick leave due to common lower back pain represent a significant healthcare cost, particularly in the patient group that does not return to work within 4 weeks, and that has a very high risk of chronic disorders. From a therapeutic perspective, too often, these patients undergo intensive treatment at too late a stage, with rather disappointing results from the long and costly treatment (the functional rehabilitation programmes offered to patients on extended sick leave in France lasted five weeks on average, most often in day admissions).

The hypothesis is that a short, more intense treatment at an earlier stage could have a beneficial effect in terms of recovery rates.

Why spa therapy?

In order to better respond to the public healthcare problems, spa centres aim to change the types of treatment offered by modernising them in two ways: by offering shorter, more intensive interventions, and including education (therapeutic education) in particular for patient populations which represent a problem in the therapeutic treatment and the treatment costs.

## I - 3 - EXPECTED RESULTS AND PROSPECTS

The study will be conducted in patients between the ages of 18 and 60 who have been on sick leave due to lower back pain for between 4 and 24 weeks.

Two groups will be studied in parallel: one will undertake a short (5-day) spa therapy intervention, during which they will receive a programme consisting of physical exercises, spa baths, psycho-behavioural treatment and therapeutic education. The other group (the control group) will receive standard care and advice typically given in these situations by general practitioners and specialists. Both groups will receive a guide on lower back pain.

While the efficacy of this programme has been demonstrated, it must be shown to lead to an increase in the return-to-work rates, improvement in pain and functional incapacity, and a quicker recovery. This would be the first treatment programme to demonstrate efficacy in this situation in France.

## I - 4 - Study feasibility

The main difficulties of this study were as follows:

- recruitment difficulties;

- follow-up difficulties.

In order to prevent these difficulties, four regions in France will be involved for this very common condition. All the research sites take part in general medical and specialist rheumatology networks in their respective regions. We also chose a local recruitment method with dissemination through the press, which proved to be effective, as well as recruitment through sick-leave agents from the Assistance Publique in these four regions, and employees from the Michelin group in the region of Auvergne.

Follow-up will be ensured by mail and by phone, in order to keep any missing data to a minimum.

Furthermore, the feasibility of the study is attested by:

- the experience of the clinical teams in the management and treatment of common lower back conditions, which will ensure sufficient recruitment;
- the experience of the methodologists in the planning and implementation of trials assessing non-pharmacological interventions;
- the experience of the spa therapy centres in the management of these patients.

## I - 5 - Expected benefits and risks

A short spa therapy programme, if shown to be effective, would allow the duration of sick leave to be reduced in patients with lower back pain.

There are no expected risks.

# II – STUDY OBJECTIVE

## II - 1 - PRIMARY OBJECTIVE

To evaluate the efficacy of a short, intensive and standardised spa therapy intervention on the return-to-work rates at 1 year for patients on sick leave from 4 to 24 weeks’ duration and for those in which an extended period of sick leave is expected.

## II - 2 - SECONDARY OBJECTIVES

To evaluate the efficacy of a short, intensive and standardised spa therapy intervention on the number of days of sick leave between 6 months and 1 year, as well as pain, function and quality of life at 1 year for patients on sick leave due to lower back pain from 4 to 24 weeks’ duration and for those in which an extended period of sick leave is expected.

# III – METHODOLOGY

## III - 1 - STUDY TYPE

This is a multi-centre, comparative, randomised, pragmatic, open trial using a modified ZELEN method.

## III - 2 - STUDY DURATION

The patients participating in this study will undergo 12 months of follow-up. The planned enrolment period is 24 months. The study is therefore expected to last 36 months. The patients will not be able to take part in a biomedical research study with therapeutic assessment likely to change the primary and secondary endpoints of the study for a duration of 12 months.

## III - 3 – RANDOMISATION ARM ASSIGNMENT

Randomisation will be stratified by site. The randomisation list will be prepared with variable block sizes. The list and block size will not be communicated to the investigators. The secrecy of the assignment will be upheld using a computerised randomisation system.

## III - 4 – EXPERIMENTAL DESIGN

**Randomised trial:**

Two patient groups will be studied: one group will be treated with a short, intensive and standardised spa therapy, and the other group will receive standard medical treatment.

The randomised, controlled trial is considered the gold standard for therapeutic assessment. Randomisation is the only method that allows comparable groups to be obtained for the known and unknown prognostic factors and screening bias to be limited.

The assessment of a spa therapy intervention raises specific methodological issues linked in particular to issues related to the choice of comparator and difficulties in achieving blinding (Boutron, JAMA 2004, Boutron, J Clin Epidemiol 2007). The planning, implementation, analysis and final written report of the results will also be done in accordance with the recommendations of the CONSORT Statement extension on non-pharmacological treatments (Boutron, Ann Inter Med 2008), and the CONSORT Statement extension on pragmatic trials (Zwarenstein M, BMJ, 2008).

**Choice of comparator**

This is a pragmatic study and, as such, the comparator will be the standard medical treatment of patients by their general practitioners, and specialists where necessary.

The content of this treatment will be systematically collected at the follow-up contacts at Months 3, 6 and 12.

**Justification of the modified Zelen method**

The primary endpoint is the return-to-work rate at 1 year. This criteria involves a certain level of subjectivity. Additionally, the secondary endpoints (pain, function and quality of life) are patient-reported criteria, which by definition are completely subjective. A recent study showed that, when there is no blinding, there is an overestimation of the treatment effect for randomised trials in which the endpoint is subjective (Wood L, BMJ 2008).

In this context, however, blinding of patients, therapists and evaluators is not possible. In order to limit the bias resulting from the lack of blinding, we propose to implement a modified Zelen method. First of all, all eligible patients will be invited to take part in a cohort study with the aim of assessing the return-to-work rate at 1 year, the duration of sick leave and the level of pain, incapacity and quality of life in patients with lower back pain. On a second occasion, after the randomisation stage, the patients randomised into the intervention group will be invited to take part in a study assessing the efficacy of a short, intensive and standardised spa therapy intervention. The patients willing to participate will sign a second consent form.

This method will prevent the risks of bias resulting from the lack of blinding. In a conventional randomised trial, the patients assigned to the control group (without intervention) are more likely to withdraw from the study, attempt to have the study treatment or change their behaviour during the endpoint assessment.

This scheme is acceptable in this context for several reasons:

1. With regard to ethics, the patients will be informed that, for scientific reasons, they will not be given details of any of the study hypotheses until the end of the study. This procedure of collecting the informed consent over two stages has already been used to assess rehabilitation, and several ethics committees have approved its use in this context (Quilty *et al.*, 2003, Rannou, National clinical research hospital protocol currently underway, Forestier R, ARD 2009).
2. With regard to methodology, the limitations of the Zelen method are the risk of contamination through refusal to undergo the intervention (short spa therapy programme). In the Thermarthrose study (Forestier R, ARD 2009), a large-scale, multi-centre trial assessed a specific treatment involving spa therapy vs standard treatment according to a Zelen method, with a 10% rate of contamination. The risk of contamination should be no higher in our study, taking into account:
   1. the lack of risk of the intervention;
   2. the lack of other treatments available;
   3. the duration of the intervention, which will be shorter;
   4. the patients who are on sick leave.

The risk of contamination in the standard-care group is highly unlikely, given that the short spa therapy programme is not currently offered outside this study.

Another risk of the Zelen method is the hypotheses being unblinded for patients. This risk is minimal, as the endpoint assessment visits (at M3, M6 and M12) will be done by post or email (or by phone, if no response is received), which prevents the patients from the two groups from being in contact with each other.

**Procedures for minimising missing data**

In order to minimise missing data, all patients will be contacted by phone and/or by email to remind them to send their assessment questionnaire in due course (3 months, 6 months and 12 months after enrolment) and, where necessary, they will be helped to fill in the questionnaire.

Furthermore, it has been demonstrated that offering clinical study patients a symbolic financial compensation improves response rates (Roberts PJ, Torgerson DJ, BMJ 2002). As such, at the end of the study, patients will receive a gift voucher when they have filled in and returned the questionnaires.

# IV – STUDY POPULATION

## IV - 1 - INCLUSION CRITERIA

- Men or women aged from 18 to 60 years (inclusive);
- Isolated lower back pain or back and radicular pain after classification of the disease (with the lower back numeric pain scale score being higher than the radicular numeric pain scale score);
- Patients on sick leave due to common lower back pain from 4 to 24 weeks’ duration and those in which an extended period of sick leave is expected;
- Medical examination carried out in advance, the results of which will be communicated to the patient;
- Patients having provided their written informed consent to take part in the study;
- Patients affiliated with a social security scheme (beneficiary or entitled).

## IV - 2 - EXCLUSION CRITERIA

- Cognitive or behavioural difficulties making the assessment impossible;
- Difficulties in comprehension and communication in the French language;
- Contraindication to undergo a short spa therapy.

## IV - 3 - REQUIRED NUMBER OF SUBJECTS

The return-to-work rate at 1 year is estimated to be 70% in the control group. The hypothesis is to reach 80% in the intervention group. With a 5% risk and a power of 80%, a total of 600 patients are planned to be enrolled: 300 patients in the short spa therapy group and 300 patients in the control group. Taking into account the risk of loss-to-follow-up, 700 patients will be enrolled.

## IV - 4 - RECRUITMENT METHOD

Patient recruitment is always difficult for this type of trial. The local/regional recruitment strategies have proven to be effective, particularly in the randomised Thermarthrose trial, a large-scale, randomised, pragmatic trial which included around 400 patients (Forestier R, ARD 2009). This type of recruitment has a logistical advantage as the patients live close to the spa centre and can go home after the intervention. It is therefore not necessary to provide accommodation for them, which has significant advantages in terms of cost and logistical organisation.

Another recruitment method that has proven to be effective is that of networks of general practitioners and specialists in the context of assessment of a Vichy spa therapy programme (Nguyen M, Br J Rheumatol 1997).

A triple recruitment method will be organised:

1. Local recruitment in the regions of Île de France, Auvergne, Languedoc-Roussillon and Rhône-Alpes, informing the patients through local media and posters in pharmacies and waiting rooms of general practitioners and specialists. The information will refer to lower back pain, but not to the short spa therapy programme. All patients that may be interested will be invited to contact a management centre, who will confirm the eligibility criteria, provide the patient with information and refer the patient to an enrolment visit. The enrolment visit will be carried out by a specially trained physician from a centre independent of the spa therapy centre. This method has already proven to be effective in trials in the area of spa therapy (Forestier R, ARD 2009).
2. Recruitment through sick-leave agents from the Assistance Publique in these four regions, and employees from the Michelin group in the region of Auvergne.
3. A more traditional patient recruitment method at the time of consultation, through the same local networks of general practitioners and rheumatologists agreeing to participate actively in this study.

# V – STUDY TREATMENT

## V - 1 – Intensive spa THERAPY

Standardisation of the intervention

A steering committee made up of rehabilitators, spa therapy physicians, rheumatologists, physiotherapists and psychotherapists from the various research sites and spa centres will be responsible for the preparation and standardisation of the intervention. This prior standardisation is essential to ensuring good reproducibility of the intervention during the study and the transfer of the intervention into routine clinical practice, if the trial demonstrates its efficacy.

Two meetings will be organised with all members of the steering committee to obtain a consensus on a therapeutic protocol, precisely describing the content and organisation of the intervention, taking into account the context and expertise of the various sites involved in the study.

Therapists

The various therapists responsible for the implementation of the intervention in this study will have a minimum of 5 years’ clinical experience. They will receive precise training in a specific meeting for the implementation of the intervention and they will receive a detailed manual describing the intervention and videos of each part of the intervention.

Content of the intervention

The patients in the intervention group will receive an intensive spa therapy consisting of the following, on a daily basis for five days:

- 2 hours of spa therapy (morning);
- 30 minutes of physical exercises (afternoon);
- 45 minutes of group therapeutic education (afternoon): two sessions dedicated to physical activity and rest (one of which with a CD), two sessions dedicated to work (one of which with a CD) and one pain-management session using a CD (45 minutes, all in the afternoon).

They will also receive a guide on lower back pain.

Treatment adherence assessment

Quantitative adherence (number and duration of sessions) and qualitative adherence (type of follow-up intervention, and method of performing the intervention) will be systematically assessed.

Tools for the future transfer of the intervention into routine clinical practice

The tools used for training the therapists and standardisation of the intervention before the start of the study will eventually be made available to clinicians for the intervention to be transferred into routine clinical practice. In particular, the intervention will be described in detail in a manual, and each part of the intervention (i.e., the exercise programme and therapeutic education) will be filmed.

Comparator

The patients in the control group will receive standard treatment with no restrictions, organised by their referring physicians, general practitioners or specialists, as well as the same guide on lower back pain given to the intervention group.

## V - 2 – ASSOCIATED TREATMENTS

The prescription of medicinal products such as analgesics and NSAIDs will be left to the investigator’s discretion. There are no prohibited treatments. Any treatment taken or likely to be taken by the patient during the study must be reported in the form of a diary, which will be given to the patient at the enrolment visit.

# VI – ENDPOINTS

## VI - 1 - PRIMARY ENDPOINT

The primary endpoint will be the return-to-work rate at 1 year. A higher return-to-work rate in the intervention arm would allow us to justify the cost of the intervention.

## VI - 2 - SECONDARY ENDPOINTS

The secondary endpoints will be as follows:

- Area under the curve of pain over the past 48 hours, assessed every fortnight using a numeric scale from 0 to 100 at 1 year;
- Functional assessment at 1 year (Quebec scale);
- Quality of Life assessment at 1 year (MOS SF-12 questionnaire);
- TWIST at 1 year (pain);
- Number of days of sick leave between 6 months and 1 year after enrolment. We have not taken into account the total number of days of sick leave as, by definition, the intervention requires the patients to be available for the duration of the treatment, which will involve sick leave.

# VII – PATIENT STUDY PLAN

## VII - 1 – D0 - ENROLMENT VISIT

Patients who may be eligible for enrolment will be referred to the Department of Physical Medicine and Rehabilitation at Cochin, Clermont-Ferrand, Montpellier or Nancy, or else to an independent assessor in Aix les Bains or Dax, in order to be enrolled in the study.

The investigator will systematically confirm the inclusion and exclusion criteria at the enrolment visit. First of all, the investigator will clearly explain to the patient the implications of participating in a cohort study with follow-up by post or email at Months 3, 6 and 12 and a pain assessment by phone every fortnight for 1 year. The patients will also be systematically informed that, for scientific reasons, they will not be given details of any of the research hypotheses until the end of the study. At the end of the study, the patients in the control group will not be able to benefit from the study results. After 1 year of follow-up, patients in the control group who are still on sick leave will no longer justify this type of intervention. If the patient agrees to participate in the cohort study, he or she will sign the initial informed consent form (ICF1).

After signing ICF1, the patients will be randomised using secure computerised randomisation software. The patients randomised into the short spa therapy programme will be offered the treatment and, if they accept, they will sign the second informed consent form (ICF2). For these patients, an enrolment form will be sent by fax to the spa therapy centre of reference, to the Clinical Research Unit (URC) and to the French Association for Spa Therapy Research (AFRETH) to plan the organisation of this treatment.

The following information will be collected in the case report form:

- - Patient contact details (surname, first name, address, email, telephone number(s));
  - Demographic parameters (age, sex);
  - Socioprofessional parameters (level of studies, profession and start date of sick leave);
  - Clinical parameters regarding the lower back pain (date of diagnosis of lower back pain, date of onset of current episode and main aetiology);
  - Assessment of pain over the past 48 hours, assessed using a numeric scale from 0 to 100;
  - Previous and ongoing treatment for lower back pain;
  - Functional indices: Quebec scale;
  - Anxiety/Depression: HAD questionnaire;
  - Quality of Life: MOS SF-12 questionnaire;
  - Fear-avoidance beliefs: FABQ questionnaire;
  - Coping strategies: CSQ questionnaire.

Following this visit, the investigator will give the Month 3, 6 and 12 follow-up self-assessment questionnaires which the patient must fill in and return by mail, from the enrolment visit for the control group and from the end of spa therapy for the intervention group.

Patients randomised into the standardised spa therapy arm will receive a start date for the intervention from the secretary at the spa therapy centre, taking into account a maximum time period of 3 months between the enrolment visit and the therapy. The planning of the stay and patient welcome will be arranged by the spa therapy centre, the choice of accommodation will be managed by the French Association for Spa Therapy Research (AFRETH) and transport passes will be managed by the Clinical Research Unit (URC).

Finally, the investigator will provide the patients with the guide on lower back pain, the patient diary containing the self-assessment questionnaires for the various evaluations, as well as the patient card mentioning the patient’s participation in the study.

## VII - 2 - INTERVENTION PROTOCOL

The patients in the intervention group will receive an intensive spa therapy consisting of the following, on a daily basis for five days (Annex III):

- 2 hours of spa therapy (morning);
- 30 minutes of physical exercises (afternoon);
- 45 minutes of group therapeutic education (afternoon): two sessions dedicated to physical activity and rest (one of which with a CD), two sessions dedicated to work (one of which with a CD) and one pain management session using a CD (45 minutes, all in the afternoon).

They will also receive a guide on lower back pain.

The spa therapy physicians will fill in and send by fax the report of any AEs/SAEs occurring during the therapy to the Clinical Research Unit (URC), at the end of each intervention.

The patients in the control group will receive standard treatment with no restrictions, organised by their referring physicians, general practitioners or specialists, as well as the same guide on lower back pain given to the intervention group.

## VII - 3 - FOLLOW-UP VISITS after the intervention

Patients will undergo follow-up at Months 3, 6 and 12. Follow-up will be done by post or email, depending on the patient’s preference, together with a telephone call if no response is received.

The following data will be evaluated:

- Professional status and return to work;
- Periods of sick leave since the previous visit (dates and number of days of sick leave);
- Assessment of pain over the past 48 hours, assessed using a numeric scale from 0 to 100;
- Lower back pain progress;
- Treatment since the previous visit;
- Adverse events collected;
- Functional indices: Quebec scale;
- Anxiety/Depression: HAD questionnaire;
- Quality of Life: MOS SF-12 questionnaire;
- Fear-avoidance beliefs: FABQ questionnaire (only at the Month 12 assessment);
- Coping strategies: CSQ questionnaire (only at the Month 12 assessment);
- Treatment satisfaction (only at the Month 12 assessment).

Pain over the past 48 hours will also be systematically evaluated once every fortnight by phone (using a numeric scale from 0 to 100). VOICE RESPONSE SYSTEM

## VII - 4 - EXITING THE TRIAL AND EARLY WITHDRAWAL

Patients may leave the study at any time and for any reason, if they decide to do so. This will have no consequences on the quality of any subsequent care that the patient will receive. Patients may also be withdrawn from the trial at the investigator’s discretion. All cases of patients withdrawing from the study must be documented.

For a patient to withdraw from the study early, the investigator must fill in the case report form up to the date of early withdrawal. The reason for the early withdrawal must be specified. Patients withdrawing from the trial cannot be enrolled again in the study. The patient’s treatment will continue in the context of routine care.

## VII - 5 - STUDY TERMINATION

The sponsor may decide to terminate the whole study early due to:

- insufficient recruitment;
- unresolved technical issues;
- express desire of the investigators;
- repeated and unjustified protocol violations.

# VIII – QUALITY CONTROL AND ASSURANCE

The research will be conducted in accordance with the standard operating procedures of the Clinical Research Unit (URC) / AP-HP.

The performance of the study at the research sites and the treatment of subjects will be done in accordance with the Declaration of Helsinki and the Good Clinical Practice guidelines in force.

## VIII - 1 - MONITORING PROCEDURES

The CRAs representing the sponsor will carry out visits to the research sites according to the follow-up schedule for patients in the protocol, the enrolments at the various research sites and the level of risk assigned to the research study. The research has a risk level A.

- Start-up visit at each site: before enrolment, for implementation of the protocol and familiarisation with the various parties involved in the biomedical research study.

- At the next visits, the case report forms will be reviewed as the research progresses by the CRAs. The principal investigator at each site, as well as the other investigators who enrol or undertake follow-up with individuals participating in the research, agree to receive visits from the CRAs at regular intervals.

In accordance with the Good Clinical Practice guidelines, the following items will be reviewed at the site visits:

- Compliance with the protocol and procedures set out for the research;
- Verification of the patient informed consent forms;
- Examination of the source documents and comparison with the data reported in the case report form in terms of accuracy, missing data, and consistency of the data according to the regulations set out by the procedures of the URC / AP-HP ?

- Closure visit: biomedical research documents, archiving.

## VIII - 2 – TRANSCRIPTION OF INFORMATION INTO THE CASE REPORT FORM

The research data will be collected using the CleanWEB electronic case report form. The data will be centralised in a server located at the Operational Services Department (DSO) of AP-HP, 67 boulevard Bessières – 75017 PARIS.

An initial version of the eCRF may be put online and tested after sending the specific study specifications by fax to the company TELEMEDICINE. Once the Coordinator, the Head of Project, the Clinical Research Unit (URC), the Data Manager and the Statistician have agreed on the final version of the eCRF, and following the release of the appropriations, and submission of the purchase order to the company TELEMEDICINE, the eCRF will come into operation.

In accordance with the Good Clinical Practice guidelines, the case report form on which the research data are transcribed must correspond to at least the following standard presentation:

- At the start of the form, the following are normally included: the title of the research study, the patient’s study reference, possibly the contact information of the individual taking part in the research (no more than three letters of their surname or the first initials of their surname and first name), randomisation number (where applicable), and inclusion and exclusion criteria in the form of a check-list, which allows subject selection to be validated with respect to the study population. At the end of the research, when the research database has been “frozen”, the eCRFs of each patient will be printed and signed by the investigator. The references of the research and of the person taking part in the research will then appear in the form of a slip on each page to allow data to be identified in all cases.
- The visit and/or sampling dates of the data transcribed must be reported in this eCRF as well as the time of the research to which they correspond.
- Results of the doses must contain the units of measurement, and even the laboratory standards in the event that these vary with the technique used.
- The following items must be included at the end of the eCRF:

- Concomitant treatments;

- Non-serious adverse events (AEs);

- End of study/Early termination;

- Outside planning, an SAE form.

All the information required by the protocol must be provided in the case report form and an explanation must be provided by the investigator for any missing information.

Information must be transferred to the case report forms as soon as it becomes available, whether clinical or paraclinical information.

Incorrect information detected in the case report forms will be replaced in the form by a registered investigator, who will log in to the software with his or her access information (user name and password). These codes are strictly personal and confidential, and under no circumstances may be passed on to third parties. They help ensure data confidentiality and authenticate the interventions. Access information is associated with an electronic signature system which validates the data entered by the investigator. Each signature is stamped with the date and time and recorded in the research Audit Trail. Signed information cannot be changed. However, the investigator may void his or her signature if he or she wishes to correct any information. Voiding a signature is also subject to stamping with the date and time.

Subject anonymity will be guaranteed by mentioning no more than the research study number, the initials of the surname and first name of the individual taking part in the research in all documents required for the study, or by erasing any personal information using a suitable method (e.g., correction fluid) from the source documents to be included in the research documentation.

The electronic data file will be declared to the CNIL in accordance with the appropriate procedure for the case.

# IX – STATISTICAL DATA MANAGEMENT

The statistical analysis will be ensured by the Prof. Ravaud Clinical Epidemiology Centre.

## IX -1- PLANNED STATISTICAL METHODS

The statistical analysis will be carried out independently by a blinded statistician from the Clinical Epidemiology Centre, using Statistical Analysis System version 9.1, under the responsibility of Dr Isabelle Boutron and Prof. Philippe Ravaud. A Statistical Analysis Plan will be prepared and validated prior to the freezing of the database and the unblinding. The Statistical Analysis Plan may be revised during the study, in order to take into account any changes made to the protocol or any changes in the performance of the study which have an impact on the originally planned statistical analyses. All versions will be kept in the study file.

## IX -1- 1- Descriptive statistics

The distribution of the parameters will be summarised according to the nature of the variable studied for the whole population and by group.

The descriptive statistics for each of the criteria will be expressed by the following parameters:

For quantitative data: sample size, mean, standard deviation of the variable, standard deviation of the mean, median, first and third quartiles, minimum, maximum and number of missing pieces of data. The 95% confidence interval of the mean will also be described.

For qualitative data: sample size and percentage associated with each of the classes. The 95% confidence interval of the percentage will also be described.

## IX -1- 2- Baseline criteria

For the criteria reported at the screening and/or enrolment visit, a descriptive analysis will be carried out on both groups. The qualitative variables will be described by their sample size, percentage and data missing by response method, and the quantitative variables will be described by their sample size, mean and standard deviation. In the event of quantitative variables with asymmetrical behaviour, these will be presented with their median and interquartile range (25^th^ percentile; 75^th^ percentile). No statistical tests comparing the treatment groups derived from the randomisation will be performed.

## IX -1- 3- Early withdrawals from the study

Subjects withdrawing from the study early and the reason for this will also undergo a descriptive analysis by group and for the total population.

## IX -1- 4- Compliance and duration of the study

The patient follow-up parameters will be analysed for each treatment group and for the total population: total follow-up duration, compliance.

## IX -1- 5- Primary endpoint analysis

An intention-to-treat analysis will be carried out, i.e., all patients randomised will be analysed in their randomisation arm, regardless of the treatment received and any missing information. The bilateral tests will be carried out at the 5% threshold. The return-to-work rate percentage will be compared using the Chi-squared test.

## IX -1- 6- Secondary analyses

A linear mixed effects regression model will be used to compare the variations between the groups of the quantitative secondary endpoints (number of days of sick leave, number of periods of sick leave, pain, Quebec scale, MOS SF-12, HAD, FABQ and CSQ). The variable to be studied will therefore be the difference between the randomisation value (D0) and that of interest (1 year), as well as the difference between the randomisation visit and those of the interim visits (3 months and 6 months). The difference in the Δ between the groups will be modelled with the following fixed effects: the group variable (2 modalities), the enrolment value as a quantitative covariate (centred variable), the visit variable (3 modalities) and the interaction between the time and the visit, allowing us to estimate the treatment effect at each visit. The model will therefore allow us to compare the adjusted means (to the baseline value and the values of the interim visits) of the variations between the final visit and the enrolment visit (as well as between the interim visits and the enrolment visit). A random effect on the patient will be added to this model in order to take into account the correlation induced by the repetition of the measurements performed in the same patient. This linear mixed model for repeated measurements (MMRM) allows the assessments performed at each visit to be taken into account, as well as the randomly missing data (the model will also integrate the endpoint values at the different visits, enrolment variables which are potentially predictive of the missing data). The area under the curve of pain between enrolment and 1 year, assessed every week, will be calculated using the trapezoidal rule and compared using the non-parametric Mann–Whitney U test. The Q-TWIST will be calculated from the percentage of time spent by a patient in various states (percentage of time spent in inactivity, time spent with acceptable pain (less than 3 on a scale from 0 to 10 which corresponds to the PASS), time spent with pain greater than 3). The values assigned to these states will be defined at the time of the blind review of the data. The means will be compared using the non-parametric Mann–Whitney U test.

## IX -1- 7- Tolerance

Adverse events

Adverse events will be described per treatment group according to their MedDRA codes (SOC and PT) and according to their intensity and imputability to the treatment. The proportion of subjects with at least one adverse event will be compared between the groups using the Chi-squared test or Fisher’s exact test.

## IX -2 - STATISTICAL CRITERIA FOR TERMINATING THE RESEARCH

None

## IX -3 - METHOD FOR TAKING INTO ACCOUNT MISSING, UNUSED AND INVALID DATA

In the event of missing data regarding the primary endpoint, the method of maximum bias will be used so as not to favour the hypotheses to be tested. The missing values in the group to be tested will be replaced by failures, while in the reference group, they will be replaced by successful cases, and vice versa.

## IX - 4- CHOICE OF INDIVIDUALS TO BE INCLUDED IN THE ANALYSES

Adults (between the ages of 18 and 60), either male or female, with isolated or lower back pain or back and radicular pain after classification of the disease, on sick leave from 4 to 24 weeks, and who meet the inclusion criteria of the study.

## IX - 5 - DESCRIPTION OF PROTOCOL DEVIATIONS

The blind review of the data will include the list of individual data as well as the list of deviations, providing the level of deviation for each subject (“no deviation”, “minor deviation” or “major deviation”). A descriptive analysis of protocol deviations will be presented by product group.

# X – SAFETY ASSESSMENT

## X - 1 - DESCRIPTION OF THE SAFETY ASSESSMENT PARAMETERS

**Adverse event**

Any harmful manifestation occurring in an individual taking part in a biomedical research study, regardless of whether or not the manifestation is related to the research.

**Adverse event in a research study not involving a product mentioned in Article L. 5311-1** (medicinal products, biomaterials and medical devices, in vitro diagnostic medical devices, labile blood products, organs, tissues, cells and products of human or animal origin, and cellular products for therapeutic purposes).

Any adverse event due to the research.

**Serious adverse event or effect**

Any adverse event or effect that leads to death, is life-threatening for the individual taking part in the research, involves hospitalisation or an extended hospital stay, causes significant or permanent incapacity or disability, or leads to a congenital abnormality or malformation.

**New fact**

Any new safety information that may lead to a reassessment of the risk-benefit ratio of the study, or which may be enough to consider making changes to the performance of the research.

## X - 2 - Adverse events

The adverse events that may occur during the study are mainly related to the treatments used.

- Main adverse events related to the education programme
  - No known or reported evidence on this subject
- Main adverse events related to the exercise programme
  - Increase in pain related to exercise
  - Muscle, ligament and joint injuries related to exercise
  - Decompensation of arterial or cardiac condition during exercise

## X - 3 - SERIOUS ADVERSE EVENT MANAGEMENT PROCEDURE

As this is a biomedical research study classified as “risk A”, i.e. for which there is a negligible **additional risk expected from the research**, no serious adverse events are expected through the course of the research.

Furthermore, there cannot be any suspected unexpected serious adverse reactions (SUSARs) related to the research.

In these conditions:

- The investigator will not be expected to declare any serious adverse events occurring during the research to the sponsor. Should such events occur, these will be related to the patient’s condition or their therapeutic treatment in the context of care, and will not be related to the research.

- It is not considered necessary to include a serious adverse event reporting form or an adverse event classification table with the protocol.

- It is not considered necessary to create an independent monitoring committee.

## X - 4 - STEERING COMMITTEE

The committee will be made up of the clinicians who initiated the project, the biostatistician in charge of the project, the sponsor’s representatives and the Clinical Research Unit (URC) appointed for this study.

It will define the general organisation and performance of the research and will coordinate the information. It will initially determine the methodology and decide over the course of the research what action must be taken in unforeseen situations, and will monitor the progress of the research, particularly with regard to tolerance and adverse events.

# XI – RIGHT TO ACCESS THE INFORMATION AND SOURCE DOCUMENTS

Individuals with direct access in accordance with the legislative and regulatory provisions in force, in particular Articles L.1121-3 and R.5121-13 of the French Public Health Code (e.g., researchers, individuals in charge of quality control, monitors, clinical research assistants, auditors and all individuals collaborating in clinical trials) will take all the necessary precautions in order to ensure the confidentiality of the information related to the investigational medicinal products, trials, individuals taking part in the research, and in particular any information involving their identity, as well as the results obtained. The data collected by these individuals through the course of quality controls or audits will then be made anonymous.

# XII – LEGAL AND ETHICAL ASPECTS

The sponsor is defined by Law 2004-806 of 9 August 2004. Before beginning the research, each investigator must provide the sponsor’s representative in the research with a signed and dated copy of their curriculum vitae, which must include their French National Medical Council registration number.

## XII - 1 - REQUEST FOR AUTHORISATION FROM THE ANSM

Before beginning the research, the sponsor must submit an authorisation request file to the competent authority (the ANSM, formerly Afssaps). The competent authority, as defined in Article L. 1123-12, makes decisions related to the safety of individuals taking part in a biomedical research study, taking into account the safety and quality of the products used during the research in accordance with the regulations in force, where applicable, their condition of use and the safety of individuals with regard to procedures carried out and the methods used, as well as the planned methods of patient follow-up.

## XII - 2 - REQUEST FOR ETHICS COMMITTEE OPINION

In accordance with Article L.1123-6 of the French Public Health Code, the sponsor must submit the research protocol to an Ethics Committee. The committee’s opinion will be reported to the competent authority by the sponsor before the research begins.

## XII - 3 - AMENDMENTS

The sponsor must be informed of any planned changes to the protocol by the principal investigator.

Amendments must be classified as substantial or non-substantial.

A substantial amendment is an amendment which may, in one way or another, change the guarantees given to the individuals taking part in the biomedical research (change in inclusion criteria, extension of enrolment period, participation of new sites, etc.).

Once the research has started, any substantial amendments on the sponsor’s initiative must receive a favourable opinion from the Ethics Committee and authorisation from the competent authority prior to being implemented. In this case, where necessary, the committee will ensure that a new consent form is duly collected from individuals participating in the research.

Moreover, any extension to the research (radical change in the treatment regimen or populations included, extension of treatments and/or therapeutic procedures not originally foreseen in the protocol) must be considered as a new research study.

Any substantial amendment must be submitted by the sponsor, after payment of the corresponding fee, for authorisation from the ANSM and/or for the Ethics Committee’s opinion.

## XII - 4 - CNIL DECLARATION

The law provides that the declaration of the electronic file with the personal data collected for the research must be prepared before the effective start of the research.

## XII - 5 - Information sheet and consent form

Written consent must be collected from any individual participating in the research before any procedures related to the biomedical research are performed.

Within the context of this study, the patients will be enrolled at the visit with the rheumatologist or the rehabilitation physician (enrolment visit). During this visit, the patient will be informed of all of the study-related information. At the end of the enrolment visit, if the patient meets all the eligibility criteria and agrees to participate in the study, he or she will provide the physician with the signed consent form.

## XII - 6 - FINAL REPORT ON THE RESEARCH

The final report on the research will be drafted by the principal investigator in collaboration with the biostatistician for this study. This report will be submitted to each of the investigators for their opinion. Once a consensus has been reached, the final version must be approved with the signature of each of the investigators and sent to the sponsor as soon as possible after the effective end of the study. A report prepared in accordance with the competent authority reference plan must be sent to the competent authority and to the Ethics Committee within one year after the end of the study. The end of the study is understood to be the last follow-up visit of the last subject enrolled. This period is set at 90 days if the research is terminated early.

# XIII – DATA PROCESSING AND STORAGE OF DOCUMENTS AND DATA RELATING TO THE RESEARCH STUDY

The documents from a research study falling under the scope of the law on biomedical research must be archived by all the parties for a period of 15 years after the end of the research (see GCP, chapter 8: essential documents).

This indexed archive consists of:

- Copies of the ANSM authorisation letter and the mandatory opinion from the Ethics Committee;
- Successive versions of the protocol (identified by the version number and date);
- Letters of correspondence with the sponsor;
- The consent forms signed by the subjects in a sealed envelope (in the case of minor subjects, these are signed by their parents or guardians) with the corresponding list or enrolment register;
- The complete and validated case report form for each subject enrolled;
- Any specific annexes to the study;
- The final study report from the statistical analysis and the quality control of the study (sent in duplicate to the sponsor);
- Certificates from any audits performed during the course of the research;

The database that gave rise to the statistical analysis must also be archived by the head analyst (hard copy or electronic copy).

# XIV – INSURANCE AND SCIENTIFIC COMMITMENT

## XIV - 1 - INSURANCE

AFRETH is the sponsor of the research. In accordance with the law on biomedical research studies, it has taken out an insurance policy with the company XX for the full duration of the research, guaranteeing its own civil liability as well as that of any intervening parties (physicians or staff involved in conducting the research) (Law no. 2004-806, Art. L.1121-10 of the French Public Health Code).

AFRETH reserves the right to interrupt the research at any given time for medical or administrative reasons. If this occurs, the investigator will be notified.

## XIV - 2 - SCIENTIFIC COMMITMENT

Each investigator undertakes to comply with the obligations of the law and to conduct the research in accordance with the GCP guidelines, complying with the principles set forth in the Declaration of Helsinki in force. To this end, a copy of the scientific commitment, dated and signed by the principal investigator of each clinical department of a participating site, will be provided to the sponsor’s representative.

A delegation of tasks form will be filled in, dated and signed by all the collaborators in the research.

# XV – RULES REGARDING PUBLICATION

# XVI – LIST OF ANNEXES

**Annex 1**: Bibliography references

**Annex 2**: Investigators and Associate Teams

**Annex 3**: Standardised therapy protocol

# ANNEX I – BIBLIOGRAPHY REFERENCES

- Andersson GB. Epidemiological features of chronic low-back pain. Lancet 1999;354:581-5.
- Anema JR, Steenstra IA, Bongers PM, de Vet HC, Knol DL, Loisel P, van Mechelen W. Multidisciplinary rehabilitation for subacute low back pain: graded activity or workplace intervention or both? A randomized controlled trial. Spine 2007;32:291-8
- Boutron I, Tubach F, Giraudeau B, Ravaud P. Blinding was judged more difficult to achieve and maintain in nonpharmacologic than pharmacologic trials. Clin Epidemiol 2004;57:543-50.
- Boutron I, Guittet L, Estellat C, Moher D, Hróbjartsson A, Ravaud P. Reporting methods of blinding in randomized trials assessing nonpharmacological treatments. PLoSMed 2007;4:e61.
- Boutron I, Moher D, Altman DG, Schultz KF, Ravaud P, CONSORT Group. Extending the CONSORT statement to randomized trials of nonpharmacologic treatment: explanation and elaboration. Ann Int Med 2008;148:295-309.
- Coudeyre E, TubachF, Rannou F, Baron G, Coriat F, Brin S, Revel M, Poiraudeau S. Effect of a simple information booklet on pain persistence after an acute episode of low back pain: a non-randomized trial in a primary care setting. PLoS One 2007;2:e706.
- Druss BG, Rosenheck RA, Sledge WH. Health and disability costs of depressive illness in a major US corporation. Am J Psychiatry 2000;157:1274-8.
- Forestier R, Desfour H, Tessier JM, Françon A, Foote AM, Genty C, Rolland C, Roques CF, Bosson JL. Spa therapy in the treatment of knee osteoarthritis: a large randomised multicentre trial. Ann Rheum Dis 2010;69:660-5.
- Hanson TH, Hansson EK. The effects of common medical interventions on pain, back function, and work resumption in patients with chronic low back pain. Spine 2000;25;3055-64.
- Karjalaïnen KA, Malmivaara A, Van Tulder M, Roine Risto, Jauhiainen M, Hurri H, Koes. Multidisciplinary biopsychosocial rehabilitation for subacute low back pain among working age adults. Cochrane Database of Systematic Reviews 2003, DOI: 10.1002/14651858.
- Magnussen L, Nilsen S, Raheim M. Barriers against returning to work-as perceived by disability pensioners with back pain: a focus group based qualitative study. Disabil Rehabil 2007;29:191-7.
- Menezes L, Maher C, McAuley J, Hancock M, Herbert R, Refshauge K, Henschke N. Prognosis for patients with chronic low back pain: inception cohort study. BMJ 2009;339:b3829.
- Nguyen M, Revel M, Dougados M. Prolonged effects of 3 week therapy in a spa resort on lumbar spine, knee and hip osteoarthritis: follow-up after 6 months. A randomized controlled trial. Br J Rheumatol 1997;36:77-81.
- Quilty B, Tucker M, Campbell R, Dieppe P. Physiotherapy, including quadriceps exercises and patellar taping, for knee osteoarthritis with predominant patello-femoral joint involvement: randomized controlled trial. J Rheumatol 2003;30:1311-7.
- Poiraudeau S, Rannou F, Le Henanff A, Coudeyre E, Rozenberg S, Huas D, Martineau C, Jolivet-Landreau I, Revl M, Ravaud P. Outcome of subacute low back pain: influence of patients' and rheumatologists' characteristics. Rheumatol 2006;45:718-23.
- Rannou F, et al. Efficacité thérapeutique d’un programme personnalisé de rééducation fonctionnelle dans la Sclérodermie Systémique. SCLEREDUC. Un PHRC national en cours.
- Reme SE, Hagen EM, Eriksen HR. Expectations, perceptions, and physiotherapy predict prolonged sick leave in subacute low back pain. BMC Musculskeletal Dis 2009;10:139.
- Roberts PJ, Roberts C, Sibbald B, Torgerson DJ. Increasing response rates to postal questionnaires. Effect of incentives on response rates must be considered. BMJ 2002;325:444.
- Savigny P, Watson P, Underwood M; Guideline Development Group. Early management of persistent non-specific low back pain: summary of NICE guidance. BMJ 2009; 338:b1805.
- Schonstein E, Kenny DT, Keating J, Koes BW. Work conditioning, work hardening and functional restoration for workers with back and neck pain. Cochrane Database Syst Rev 2003;(1):CD001822.
- Waddell G, Burton AK. Occupational health guidelines for the management of low back pain at work: evidence review. Occup Med 2001;51:124-35.
- Wood L, EggerM, Gluud LL, Schulz KF, Jüni P, Altman DG, Martin RM, Wood AJ, Sterne JA. Empirical evidence of bias in treatment effect estimates in controlled trials with different interventions and outcomes: meta-epidemiological study. BMJ 2008; 336:601-5.
- Zwarenstein M, Treweek S, Gagnier JJ, Altman DG, Tunis S, Haynes B, Oxman AD, Moher D; CONSORT group; Pragmatic Trials in Healthcare (Practihc) group. Improving the reporting of pragmatic trials: an extension of the CONSORT statement. BMJ 2008;337:a2390.

# ANNEX II – INVESTIGATORS AND ASSOCIATE TEAMS

| Site no. | Investigators | Hospital and Department | Associated spa therapy centre |
| --- | --- | --- | --- |
| 01 | Prof. Serge Poiraudeau | Department of Physical Medicine and Rehabilitation of the Musculoskeletal System and Spinal Disorders  Hôpital Cochin  27 rue du Faubourg Saint Jacques  75014 PARIS | St Amand les Eaux  Route de la Fontaine Bouillon  BP 80 108  59230 St Amand les Eaux |
| 02 | Prof. Emmanuel Coudeyre | Department of Physical Medicine and Rehabilitation  CHU Clermont-Ferrand, Hôpital Nord,  61 rue de Chateaugay  63 118 Cébazat | Etablissement Thermal de Royat  Place Allard - BP 53  63130 ROYAT CHAMALIERES |
| 03 | Dr Arnaud Dupeyron  Dr Isabelle Lafont? | Department of Physical Medicine and Rehabilitation  Hôpital Lapeyronie  CHU Montpellier | Balaruc les Bain  BP 45  34540 BALARUC LES BAINS |
| 04 | Dr Ygal Attai  Dr Romain Forestier | General Practitioner  3 place de la Libération  73000 Chambéry | Les Thermes Nationaux  Place Maurice Mollard - BP 349  73103  Aix les bains |
| 05 | Dr Jean-Max Tessier  Dr Charles Cowan? | Hôpital thermal de Dax | Etablissement de cures thermales de Dax  1, rue Labadie - BP 323  40107 Dax |
| 06 | Prof. Isabelle Chary-Valckenaere | Rheumatology Department  CHU Nancy  Rue du Morvan  54511 VANDOEUVRE Cedex | Amneville les Thermes |

# ANNEX III – Standardised spa therapy protocol

1. Spa therapy programme (2 hrs/day)
2. Exercise programme (½ hr)
3. Group therapeutic education (45 mins)
   - Physical activity and rest
   - Work
   - Pain management
4. Back guide

**Distribution and content of the five sessions:**

| Times | **1^st^ session** | **2^nd^ session** | **3^rd^ session** | **4^th^ session** | **5^th^ session** |
| --- | --- | --- | --- | --- | --- |
| **2 hrs**  **morning** | Spa therapy | Spa therapy | Spa therapy | Spa therapy | Spa therapy |
| Rest | | | | | |
| **½ hr**  **afternoon** | Exercise | Exercise | Exercise | Exercise | Exercise |
| **45 mins**  **afternoon** | TE | TE  with CD | TE | TE  with CD | TE  with CD |

TE: Therapeutic education

1. **Spa therapy programme (2 hrs/day)**

Staff in charge: Spa therapy physician and spa therapy technician

The patient is first of all assessed by the spa therapy physician.

Session plan:

- Hydrojet baths (or whirlpools): six 20-minute sessions in water at 38°.
- Massage shower: 10 minutes of manual massage underwater at 38°. The massage is followed by a 3-minute shower at 38°, performed remotely by the masseur. The strength of the massage and the jet must be specified by the spa therapy physician (gentle if the patient is experiencing intense pain and is apprehensive, more powerful if not).
- Directed mobilisation pool at 35°: 10 minutes of free bathing, 15 minutes of directed mobilisation by a qualified technician. This will be in the form of generic movements involving the entire spine and joints, as currently performed at these centres. Lumbar movements must include lumbar stretches.
- The spa therapy must be ended with the application of spa mud. The spa therapy physician must specify whether it will be applied directly to the skin or wrapped in a cloth (poultice). The temperature of the spa mud will be between 45° and 50° and the sessions will last between 15 and 20 minutes.

1. **Exercise programme (½ hour/day)**

Staff in charge: Physiotherapist

**2.1. Isometric strengthening of the spine (10 mins)**

- Lie with a cushion under the stomach, hands under the forehead and elbows apart.
- Lift the upper part of the torso, with the head aligned with the back.
- Hold for 6 seconds, repeat 5 times, resting for 6 seconds between each exercise.
- You can carry out 10 series of 5.

**2.2. Isometric strengthening of the abdomen (10 mins)**

- Lie on the back, arms crossed on the chest, legs bent at 90 degrees, with the feet flat and slightly apart.
- Lift the back to the middle of the shoulder blades while exhaling, with the head aligned with back.
- Hold for 6 seconds, repeat 5 times, resting for 6 seconds between each exercise.
- You can carry out 10 series of 5.

**2.3. Spinal-psoas-abdominal isometric co-contraction (10 mins)**

- Sit at a table, with the forearm flat and the feet flat and slightly apart.
- While exhaling, raise yourself up, rest the forearms on the table and lift a knee against the table top.
- Hold for 6 seconds, repeat 5 times, resting for 6 seconds between each exercise.
- You can carry out 10 series of 5.

1. **Group therapeutic education (45 mins/day)**

Staff in charge: Trained nurse or technician.

We will provide you with a CD on physical activity and rest, a second CD on work and a third CD on relaxation and pain management.

We advise you to put on the CDs for sessions 2, 4 and 5 (for a duration of 15 to 20 mins) and then discuss the messages transmitted in the CDs with the patients. Groups can include 4 to 6 patients.

**3.1. Physical activity and rest (1^st^ session)**

- If you remain inactive:
  - You may become stiff
  - Your muscles may become weak
  - Your skeleton may become fragile
  - Your overall physical condition may deteriorate
  - You may feel depressed
  - You may feel increasingly worse
  - It will be increasingly difficult for you to resume normal daily activities for your age (activities at home, leisure, work, etc.)
- If you remain active:
  - Your muscles will become stronger
  - You will become relaxed
  - Your skeleton will be strengthened
  - You will feel better, both physically and mentally
  - You will be fitter than before
  - Your body will produce chemicals that reduce your pain
  - Your pain will eventually subside
- Staying active means:
  - Keeping moving
  - Not remaining in one position over a long period of time
  - Moving before becoming stiff
  - Moving a little more and more quickly each day
  - Not giving up your normal activities, but changing the way you do them
  - Staying active will help you recover more quickly, and can prevent a future episode of lower back pain.
- Do not forget:
  - Resting for more than two days is not recommended, even in the event of a very painful episode.
  - Continuing to work will help you recover more quickly.
  - Adapted physical exercises performed daily for at least 20 minutes leads to fewer painful episodes, which gradually become less severe.
  - Muscle groups that have been resting for even a few days quickly lose their strength, and when they become active again, they can cause additional pain.
  - No physical activities are prohibited when you have lower back pain.

**3.2. CD on physical activity and rest (2^nd^ session)**

**3.3. Work (3^rd^ session)**

- - Resting for more than two days is not recommended.
  - The longer the initial sick leave period lasts, the higher the risk of the pain becoming chronic.
  - No work activities are prohibited when you have lower back pain.
  - Your back is painful but not fragile. Your condition is not at risk of worsening if you return to work.
  - If you experience a relapse of the pain when you return to work, you can speak to your occupational physician.
  - Having a satisfying job reduces the risk of lower back pain becoming chronic.

**3.4. CD on work (4^th^ session)**

**3.5. CD on pain management (5^th^ session)**

- - Setting short-term and long-term goals according to needs.
  - Self-encouraging, with positive thoughts.
  - Improving physical condition.
  - Thinking about positive things when you feel the pain.
  - Self-controlling: improving analysis and management of pain.
  - Seeking the support of others (family, friends, professional help), without becoming a bystander.
  - Learning to distance yourself from pain.
  - Relaxing (using the abdominal breathing method, for example).

1. **Back guide**

In most cases, the individuals themselves can manage their back pain. This guide provides suitable advice for managing pain, preventing it from being excessively uncomfortable on a daily basis and helping you recover more quickly. This guide is based on the most recent medical information.

The guide is currently being validated by Prof. Emmanuel Coudeyre.
